# Systematic review of the cost‐effectiveness of preoperative antibiotic prophylaxis in reducing surgical‐site infection

**DOI:** 10.1002/bjs5.45

**Published:** 2018-04-14

**Authors:** J. Allen, M. David, J. L. Veerman

**Affiliations:** ^1^ Queensland Audit of Surgical Mortality, Royal Australasian College of Surgeons Brisbane Queensland Australia; ^2^ School of Public Health University of Queensland Brisbane Queensland Australia; ^3^ School of Medicine Griffith University Southport Queensland Australia; ^4^ School of Medicine and Public Health University of Newcastle Callaghan New South Wales Australia; ^5^ Cancer Council NSW Woolloomooloo New South Wales Australia

## Abstract

**Background:**

Surgical‐site infections (SSIs) increase the length of hospital admission and costs. SSI prevention guidelines include preoperative antibiotic prophylaxis. This review assessed the reporting quality and cost‐effectiveness of preoperative antibiotics used to prevent SSI.

**Methods:**

PubMed, Web of Science, Cumulative Index to Nursing and Allied Health Literature, Index of Economic Articles (EconLit), Database of Abstracts of Reviews of Effect (including the National Health Service Economic Evaluation Database) and Cochrane Central databases were searched systematically from 1970 to 2017 for articles that included costs, preoperative antibiotic prophylaxis and SSI. Included were RCTs and quasi‐experimental studies conducted in Organisation for Economic Co‐operation and Development countries with participants aged at least 18 years and published in English. Two reviewers assessed eligibility, with inter‐rater reliability determined by Cohen's κ statistic. The Consolidated Health Economic Evaluation and Reporting Standards (CHEERS) and modified Drummond checklists were used to assess reporting and economic quality. Study outcomes and characteristics were extracted, and incremental cost‐effectiveness ratios were calculated, with costs adjusted to euros (2016) (€1 = US $1·25; £1 sterling = €1·28).

**Results:**

Twelve studies published between 1988 and 2014 were included from 646 records identified; nine were RCTs, two were nested within RCTs and one was a retrospective chart review. Study quality was highest in the nested studies. Cephalosporins (first, second and third generation) were the most frequent prophylactic interventions. Eleven studies demonstrated clinically effective interventions; ten were cost‐effective (the intervention was dominant); in one the intervention was dominated by the control; and in one the intervention was more effective and more expensive than the control.

**Conclusion:**

Preoperative antibiotic prophylaxis does reduce SSI, costs to hospitals and health providers, but the reporting of economic methods in RCTs is not standardized. Routinely nesting economic methods in RCTs would improve economic evaluations and ensure appropriate selection of prophylactic antibiotics.

## Introduction

Surgical‐site infections (SSIs) occur in 1–25 per cent of surgical patients, although the occurrence and severity vary^1^, ^2^, ^3^. These variations depend on the type, duration and time of day of the operation, and the time from infection onset to detection and successful treatment^1^ ^3^, ^4^, ^5^, ^6^. SSI leads to longer hospital stays and higher costs to patients, hospitals and health systems^7^, ^8^, ^9^, ^10^, ^11^. In Europe, a minimum estimate of increased health cost due to SSI in 2004 was €1·47–19·1 billion^12^, and more recently in the USA (2014) SSI was associated with double the costs compared with those for a patient without SSI^13^.

Jointly, the Centers for Disease Control and Prevention (CDC) in the USA, the National Institute for Health and Care Excellence in the UK and the World Health Organization developed SSI prevention guidelines^4^. These include several prevention measures: preoperative screening of patients and decolonization of nasal cavities, showering, hair removal, intraoperative skin preparation using chlorhexidine, preoperative prophylactic antibiotic administration (within 1 h before surgery), normothermia and body temperature regulation, use of incision drapes, administration of supplemental oxygen throughout the operation, control of the patient's glucose level, and postoperative use of surgical dressings and appropriate hand hygiene. The prevention measures may be implemented individually or as a bundle (3–5 interventions are grouped together).

Several systematic reviews have reported on aseptic skin preparation (including surgical hand asepsis, intraoperative skin antisepsis and skin preparation with chlorhexidine)^14^, ^15^, ^16^, dressings including wound edge protection devices^16^ ^17^, increased oxygen supplementation^18^, glucose control^19^ and thermoregulation^20^. Two reviews have reported on the cost‐effectiveness of the interventions^14^ ^16^ and the quality of health economic reporting^16^.

Despite the routine use of antibiotic prophylaxis, which is inexpensive^21^, ^22^, ^23^, SSIs continue to occur. This suggests that implementation of SSI prevention is suboptimal – that more can be done, and done cost‐effectively. To date, no cost‐effectiveness review of preoperative antibiotic prophylaxis has been performed, despite the existence of clinical guidelines for antibiotic prophylaxis in surgery^21^, ^22^, ^23^.

The aim of this review was to evaluate the cost‐effectiveness of preoperative antibiotic prophylaxis used to prevent SSIs, and to assess the reporting quality of clinical effectiveness and cost‐effectiveness for each study.

## Methods

### Data sources

Published studies were identified by following the Cochrane Review Group search strategy^24^, the University of York Centre for Reviews and Dissemination^25^ and the PRISMA statement^26^. Six databases were searched: the Cochrane Library (Cochrane Central), PubMed, Cumulative Index to Nursing and Allied Health Literature (CINAHL via EBSCO), Web of Science core collection, Journal of Economic Literature and the Index of Economic Articles (EconLit via EBSCO), and Database of Abstracts of Reviews of Effect (DARE, via the University of York Centre for Reviews and Dissemination, which incorporates the National Health Service Economic Evaluation Database (NHS EED)). Earlier databases were searched from 1970 (PubMed, EconLit) and others from 1994 (DARE and NHS EED), 1996 (Cochrane Central) and 1982 (CINAHL). The search of all databases was concluded on 28 June 2017.

### Search strategy

Keywords and search terms were matched with database‐specific medical subject heading (MeSH) terms or title fields. Keywords for four different themes were linked with AND (cost AND prophylaxis AND prevention AND surgical‐site infection). Full search strategies can be found in *Table S1* (supporting information). Search results were exported into EndNote^®^ version X7 (Thomson Reuters, New York, USA) and duplicates were removed. Manual screening of references from included articles was performed to identify additional publications not identified by the search.

### Selection criteria

Systematic reviews, guidelines, conference proceedings and letters were excluded. Only articles published in English and in peer‐reviewed journals were included. The studies had to define a SSI, even if it did not conform to the CDC definition^4^: an infection related to an operative procedure that occurs at or near the surgical incision within 30 days of the procedure or within 1 year if an implant is left in place. PICO (population, intervention, comparison and outcomes) were used to evaluate study eligibility. Studies were included if they were economic evaluations in RCTs or quasi‐experimental studies that compared the efficacy between different antibiotic prophylaxis regimens or placebo. Economic evaluations were defined as the comparative analysis of the costs and consequences of alternative programmes^27^. Studies were excluded if they were performed in non‐OECD (Organisation for Economic Co‐operation and Development) countries. OECD countries were defined as high‐income‐earning economies^28^, and included 31 OECD members (*Table S2*, supporting information). Other exclusion criteria were: study participants younger than 18 years of age and surgery that did not require a general anaesthetic.

### Data extraction

Data from outcomes and resource use studies were used to construct and judge the cost‐effectiveness. Two reviewers independently applied the inclusion and exclusion criteria to the eligible studies. They first screened the titles, then abstracts and finally the full text. At each step their agreement was assessed using Cohen's κ statistic with a 95 per cent c.i.^29^. Cohen's κ statistic adjusts the proportion of articles for which there is agreement by the amount of agreement expected by chance alone^29^ ^30^. Agreement strengths for Cohen's κ are defined^29^ ^30^ as: poor, κ < 0·00; slight, κ = 0·00–0·20; fair, κ = 0·21–0·40; moderate, κ = 0·41–0·60; substantial, κ = 0·61–0·80; and almost perfect, κ = 0·81–1·00.

Disagreements were resolved by discussion, and when consensus could not be reached a third reviewer acted as referee. Reasons for exclusion were documented. All eligible articles that passed the full‐text screening were included in the review.

Extracted study data were recorded in a data collection form; they included year and country of study, study design, definition of SSI, population demographics, surgical procedures, antibiotic prophylaxis (costs, dosage and mode of administration), mean hospital and patient costs, and outcome data (duration of hospital stay, mortality, incidence of SSI, bacteria identified and antimicrobial resistance).

### Reporting quality assessment

The 24‐item Consolidated Health Economic Evaluation and Reporting Standards (CHEERS) checklist^31^ was used to assess comprehensively the quality of the clinical and methodological reporting relating to title, structured abstract, methods, results, discussion, conclusion, funding and conflicts of interest. Two of the checklist items (choice of a model and assumptions) were not included as they were not applicable to any of the studies. Each of the remaining 22 items were assigned a weighted rating^16^: 0, did not report; 1, reported poorly; 2, reported well. The overall quality rating is the proportion of items reported well: high quality, 17 or more of 22 (77 per cent or above); medium/acceptable quality, 11 or more and fewer than 17 of 22 (50 per cent or above and less than 77 per cent); and low/unacceptable quality, fewer than 11 of 22 (less than 50 per cent). There is methodological reporting overlap between the CHEERS checklist and the economic quality checklist described below.

### Economic quality assessment

A modified version of the Drummond *et al*. checklist^27^ was used to assess the quality of the economic and methodological reporting. The checklist includes ten questions, of which two have subquestions. These 12 questions enabled assessment of the following elements for each study: methods used (appropriate and accurate measurement of costs and outcomes), clinical effectiveness, limitations, uncertainty, relevance, generalizability and conclusions. Answers assigned to each question could be: ‘yes’, ‘no’ or ‘not applicable’. The overall quality ratings are based on the number of questions answered as ‘yes’: high quality, nine or more of 12 (75 per cent or above); medium/acceptable quality, six or more and fewer than nine of 12 (50 per cent or more and less than 75 per cent); and low/unacceptable quality, fewer than six of 12 (less than 50 per cent).

### Incremental cost‐effectiveness ratio

When treatment effect (TE) and incremental cost‐effectiveness ratios (ICERs) were not reported, they were calculated using the study data. Treatment effect is defined as the difference between the control and intervention effect (TEc − TEi). To determine the incremental cost saving of SSIs averted, the difference in mean total cost between the intervention and control prophylaxis was divided by the treatment effect. Calculated ICER costs were then adjusted to British pounds (2016) in a two‐step process, using the Campbell and Cochrane Economics Methods Group–Evidence for Policy and Practice Information and Coordinating Centre cost converter web‐based tool^32^ ^33^. Step 1 inflates the cost from the original price year to April 2016, using a Gross Domestic Product deflator index (GDPD values), obtained from the International Monetary Fund World Economic Outlook Database GDP deflator index data set^34^. Step 2 converts the original currency to British pounds, using conversion rates based on Purchasing Power Parities for GDP (PPP values)^32^ ^33^. Using a web‐based tool, the 2016 British pound to euro conversion factor for £1 sterling is €1·28. When not stated, accepted standard practice to infer price year and/or currency^33^ was used. The price year was assumed to be either the year the study ended or the year of publication, and the original currency to be the same as that in the study setting.

## Results

**Figure 1:**
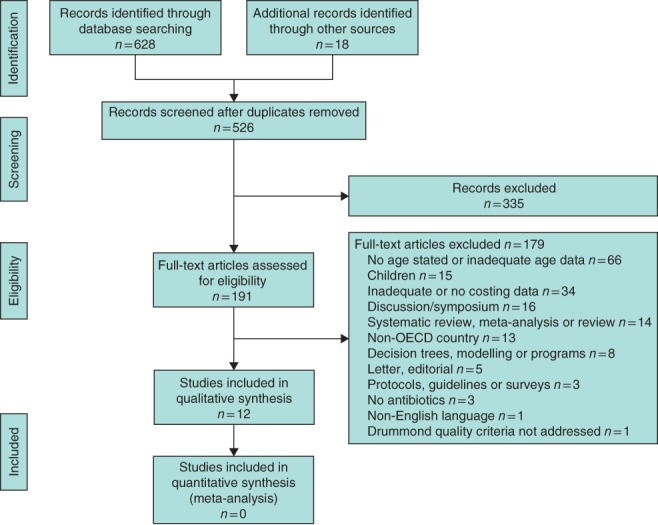
PRISMA flow diagram for the review. OECD, Organisation for Economic Co‐operation and Development

The search yielded 628 articles; 508 remained once duplicates had been removed. The remaining articles were subjected to a systematic review by two independent reviewers who applied the inclusion criteria. A further 18 articles were identified by hand‐searching. The inclusion criteria were first applied to the article titles, then abstracts and finally the full text. Cohen's κ statistic calculated for each step showed almost perfect (κ = 0·89, 95 per cent c.i. 0·80 to 0·98), substantial (κ = 0·64, 0·53 to 0·75) and moderate (κ = 0·55, 0·45 to 0·65) agreement respectively. Five full‐text articles required review by a third reviewer, and one was included. The five main reasons for full‐text exclusion were: age restriction (81 articles), inadequate or no cost data (34), discussion or symposium paper (16), systematic review (14) and studies performed in non‐OECD country (13). Twelve articles met the inclusion criteria (*Fig*. 1).

*Table* 1 provides detailed characteristics of the 12 included studies^35^, ^36^, ^37^, ^38^, ^39^, ^40^, ^41^, ^42^, ^43^, ^44^, ^45^, ^46^. These were published between 1988 and 2014 with four published after 2000^38^ ^39^, ^44^ ^46^. Nine^36–38,40–45^ were RCTs, two^39^ ^46^ were nested within an RCT and one^35^ was a retrospective chart review. Eight were conducted in Europe (Greece^35^ ^43^, Scotland^37^, UK^38^, Spain^40^, Italy^42^, Finland^45^ and the Netherlands^39^), three in the USA^36^ ^41^, ^46^ and one in Japan^44^. The studies encompassed head and neck, gynaecological, vascular, cardiothoracic, general (breast and endocrine, intestinal and colorectal, and hepatopancreatobiliary) and orthopaedic surgery. Eleven studies^35–38,40–46^ evaluated the effectiveness of preoperative prophylaxis of the antibiotic cephalosporin (either first, second or third generation). These included ‘clean’ surgery (neck dissection^35^, axillary lymph node dissection^36^, coronary artery bypass graft (CABG)^38^ ^45^, abdominal aortic or lower limb prosthetic vascular surgery^42^) and ‘clean‐contaminated’ surgery (abdominal or vaginal hysterectomy^37^ ^41^, ^43^, digestive tract resection with anastomosis^39^, colonic resection and colorectal surgery^41^ ^46^, biliary^40^ and gallbladder surgery^44^). One study^39^ evaluated selective decontamination of the digestive tract in clean‐contaminated surgery of the digestive tract with anastomosis.

**Table 1:** Characteristics of included studies

			Preoperative prophylaxis		Preoperative prophylaxis outcome measures		
Reference	Population	Follow‐up	Control	Intervention	Primary (efficacy)	Secondary (cost analysis)	Conclusion
Blair *et al*.^35^ (1995)	‘Clean’ neck dissection: 192	n.s.	No prophylaxis	Cefazolin 600 mg*	First‐generation cephalosporin; clindamycin and penicillin *versus* no antibiotic to prevent postoperative wound infection	Cost‐benefit analysis (hospital stay and cost)	No significant difference in infections. Preoperative antibiotic prophylaxis advocated. Cost‐effective
			No prophylaxis	Clindamycin 2 g*			
			No prophylaxis	Penicillin*			
			No prophylaxis	Drug name n.s.*, †			
Bold *et al*.^36^ (1998)	Axillary lymph node dissection: 178	4 weeks after surgery	Placebo (normal saline)	Cefonicid 1 g (single dose)	Second‐generation cephalosporin *versus* placebo to decrease postoperative wound complications	Cost‐benefit analysis	No significant difference in infections. Preoperative antibiotic prophylaxis advocated
Davey *et al*.^37^ (1988)	Abdominal or vaginal hysterectomy: 400	Every 3 days, then after discharge (visit week 2, phone call week 6)	Placebo (normal saline)	Cephradine 2 g (single dose)	First‐generation cephalosporin *versus* broad‐spectrum penicillin to prevent wound infection	Cost‐benefit analysis (patient, hospital and community services)	Cephradine antibiotic prophylaxis advocated in abdominal hysterectomy. Antibiotic prophylaxis questionable in vaginal hysterectomy
				Mezlocillin 5 g (single dose)			
Dhadwal *et al*.^38^ (2007)	Median sternotomy for primary CABG of at least 1 thoracic artery and at least 1 of 4 defined risk factors: 201‡ and 186§	Daily until discharge, then after discharge (week 6 and 90 days)	Cefuroxime 1·5 g (single dose), then cefuroxime 750 mg at reversal of anticoagulation, 8 and 16 h after surgery	Rifampicin 600 mg (single dose), then gentamicin 2 mg/kg + vancomycin 15 mg/kg on induction of anaesthesia. Postoperative vancomycin 7·5 mg/kg at 12, 24 and 36 h	Second‐generation¶ cephalosporin *versus* gentamicin combined with rifampicin and vancomycin to prevent sternal wound infection	Cost‐benefit analysis	Longer and broader‐ spectrum preoperative antibiotic prophylaxis advocated. Cost‐effective
Dijksman *et al*.^39^ (2012)	Intestinal resection with primary anastomosis, with or without a diverting ileostomy or closure of a temporary colostomy: 289	1 year	Placebo for 2 days before surgery, then parenteral perioperative cefuroxime 1500 mg + metronidazole 500 mg 30 min before surgery. Cefuroxime 1500 mg + metronidazole 500 mg continued 8‐hourly for 24 h	SDD (polymyxin B sulphate100 mg + tobramycin 80 mg + amphotericin B 500 mg) for 2 days before surgery and continued for at least 3 days after surgery or until normal bowel function. Parenteral perioperative antibiotic cefuroxime 1500 mg + metronidazole 500 mg 30 min before surgery. Cefuroxime 1500 mg + metronidazole 500 mg continued 8‐hourly for 24 h	Perioperative selective decontamination of digestive tract (polymyxin B sulphate with tobramycin and amphotericin B) *versus* placebo to reduce infection	Cost‐effectiveness analysis	Selective decontamination of digestive tract advocated. Cost‐effective
Garcia‐Rodriguez *et al*.^40^ (1989)	Gastroduodenal or biliary surgery with at least 1 of 11 defined risk factors: 1451	16 days	Cefoxitin 2 g (single i.v. dose), then cefoxitin 2 g 6, 12 and 18 h after surgery	Cefotaxime 1 g (single dose)	Second‐ and third‐generation cephalosporin¶ to prevent postoperative infection	Cost‐benefit analysis	Cefotaxime antibiotic prophylaxis advocated. Cost‐effective
Jones *et al*.^41^ (1987)	Obstetrics and gynaecology, gastrointestinal; orthopaedics and other (total joint replacement and open reduction of fractures) surgical procedures: 812	30 days	Cefotaxime 1·0 g (slow i.v. bolus after anaesthesia but 30 min before incision). Additional cefotaxime 1·0 g given during surgery if procedure duration 2 h or more. For bowel surgery, standard bowel preparation before prophylaxis	Cefoperazone 1·0 g (slow i.v. bolus after anaesthesia but 30 min before incision). For bowel surgery, standard bowel preparation before prophylaxis	Two third‐generation cephalosporins to prevent perioperative infection	Cost containment	Both cefoperazone and cefotaxime antibiotic prophylaxis advocated. Both cost‐effective
Marroni *et al*.^42^ (1999)	Abdominal aortic or lower limb prosthetic vascular surgery: 238	Daily until discharge, then after discharge (3 monthly for 1 year, then at 24 months)	Cefazolin 2 g (single i.v. dose)	Teicoplanin 400 mg (single dose)	Efficacy and tolerability of first‐generation cephalosporin and a glycopeptide to prevent postoperative infection	Cost‐benefit analysis	Cefazolin antibiotic prophylaxis advocated. Cost‐effective
Matkaris *et al*.^43^ (1991)	Abdominal hysterectomy: 200	4–5 days if no SSI, otherwise kept in hospital until infection resolved	No prophylaxis	Ceftriaxone 2 g (single dose). Additional dose if postoperative infection	Efficacy and safety of three third‐generation cephalosporins to prevent postoperative infection	Cost‐benefit analysis	Single dose of any of the three antibiotic prophylaxes advocated. Cefotaxime was most cost‐effective
				Cefotaxime 2 g (single dose). Additional dose if postoperative infection			
				Ceftazidime 2 g (single dose). Additional dose if postoperative infection			
Matsui *et al*.^44^ (2014)	Laparoscopic cholecystectomy for gallbladder stones or polyps: 437	8 days after surgery in outpatient setting	No prophylaxis	Cefazolin 1 g (3 doses before skin incision, then 12 and 24 h after surgery). Additional cefazolin 1 g in theatre if duration of surgery more than 3 h	First‐generation† cephalosporin to reduce postoperative complications, including SSI and distant infection	Cost‐ effectiveness analysis	Antibiotic prophylaxis advocated. Cost‐effective
Sisto *et al*.^45^ (1994)	CABG: 551	Daily until discharge (10–12 days) or to another hospital (6–7 days)	Ceftriaxone 2 g (single dose)	Cefuroxime 1·5 g (single dose), then cefuroxime 1·5 g (8‐hourly to end of postoperative day 2)	Efficacy and side‐effects of single‐dose third‐generation cephalosporin *versus* multiple doses of second‐generation cephalosporin to prevent postoperative infection	Cost‐benefit analysis	Efficacy of ceftriaxone and cefuroxime equivalent. Ceftriaxone cheaper and simpler to use
Wilson *et al*.^46^ (2008)	Colorectal surgery: 672#	4 weeks after surgery	Ertapenem 1 g (single dose)	Cefotetan 2 g (single dose)	Preoperative prophylaxis of second‐generation cephalosporin and a β‐lactam to reduce postoperative infectious complications	Cost‐benefit analysis	Ertapenem antibiotic prophylaxis advocated. Cost‐effective

* Prophylactic antibiotic dose not stated;

† antibiotic trade name or generation of the cephalosporin not stated;

‡ intention‐to‐treat data for antibiotic efficacy;

§ per‐protocol data for costs^38^;

¶ blinding not stated;

# per‐protocol data. n.s., Not stated; CABG, coronary artery bypass graft; SDD, selective decontamination of digestive tract; i.v., intravenous; SSI, surgical‐site infection. A more detailed version of this table is available as *Table S3*, supporting information^47,48^.

### Quality assessment of reporting

The reporting quality of most of the studies was low to moderate using the CHEERS statement checklist^31^ (*Table* 2; *Table S4*, supporting information). Only one study^39^ had a high reporting quality for 18 of the 22 items. Three studies^37^, ^38^, ^39^ reported economic evaluations in their titles. In most studies the objectives, methods (settings, populations and comparators) were well reported^35^, ^36^, ^37^, ^38^, ^39^ ^41^, ^43^, ^44^, ^45^, ^46^, although time horizons and discounting were poorly reported^35^ ^37^, ^38^ ^40^, ^41^, ^42^, ^43^, ^44^ ^46^. Overall the results were poorly reported, including study parameters, incremental costs and characterization of uncertainty and heterogeneity^36^, ^37^, ^38^, ^39^, ^40^, ^41^, ^42^, ^43^, ^44^, ^45^, ^46^. Discussion around the individual study findings, their limitations and generalizability was also of poor quality^37^ ^40^, ^41^, ^42^, ^43^, ^44^, ^45^, ^46^. Source of funding and conflict of interest was poorly reported: four^35^ ^36^, ^41^ ^44^ reported funding and two^38^ ^44^ reported conflict of interest. Only one^44^ of these studies reported on both funding and conflict of interest.

**Table 2:** CHEERS checklist summary of reporting quality

		No. of studies reporting (*n* = 12)		
	Questions	Not reported	Poorly reported	Well reported
Title and abstract	Title	6	3	3
	Abstract	0	6	6
Introduction	Background and objectives	0	2	10
Methods	Target population and subgroups	0	3	9
	Setting and location	0	4	8
	Study perspective	0	5	7
	Comparators	0	5	7
	Time horizon	3	6	3
	Discount rate	12 n.a.	0	0
	Choice of health outcomes	2	7	3
	Measurement of effectiveness	2	7	3
	Measurement and valuation of preference‐based outcomes	1 n.a.	7	4
	Estimating resources and costs	1 n.a.; 1	7	3
	Currency, price date and conversion	5	6	1
	Choice of model	12 n.a.	0	0
	Assumptions	12 n.a.	0	0
	Analytical methods	0	11	1
Results	Study parameters	12	0	0
	Incremental costs and outcomes	10	0	2
	Characterizing uncertainty	9	1	2
	Characterizing heterogeneity	3	8	1
Discussion	Study findings, limitations, generalizability and current knowledge	0	9	3
Other	Source of funding	8	0	4
	Conflict of interest	10	0	2

n.a., Not applicable.

### Clinical effectiveness of antibiotic prophylaxis, length of hospital stay and mortality

All studies included a definition for postoperative SSI (*Table* 3). Four studies^38^ ^40^, ^42^ ^46^ used several variations of recognized definitions: the National Nosocomial Infections Surveillance^54^, ^55^, ^56^, variations of the CDC definition^50^ ^53^ and the National Research Council definition^50^ ^52^. The definition used by Blair and colleagues^35^ was developed by Johnson and co‐workers^49^ in 1984, and the definition reported by Dijksman *et al*.^39^ was that of Rommes *et al*.^51^, used in the nested study of Roos and colleagues^47^.

**Table 3:** Evidence of efficacy of preoperative prophylactic antibiotics

			Preoperative prophylaxis		Sample size			Postoperative infections		
Reference	Surgical procedure	Definition of postoperative infection	Control	Intervention	Total (M : F)	Control*	Intervention*	Control*	Intervention*	*P*
Blair *et al*.^35^ †	Neck dissection	Wound infection: based on wound grading scale developed by Johnson *et al*.^49^	No prophylaxis	Cefazolin 600 mg	192 (139 : 53)	99 (51·6)	58 (30·2)	10 (10·0)	3 (3·3)	0·08
				Clindamycin 2 g			13 (6·8)			
				Penicillin			17 (8·6)			
				Drug n.s.			5 (2·6)			
Bold *et al*.^36^	Axillary lymph node dissection	Infection of surgical wound in the absence of any other site of infection	Placebo (normal saline)	Cefonicid 1 g	178 (24 : 154)	90 (50·6)	88 (49·4)	12 (13·0)	5 (6·0)	0·08‡
Davey *et al*.^37^ §	AH or VH	Infected wound; pelvic infection	Placebo (normal saline)	Cephradine 2 g	400 (0 : 400)	AH 102 (25·5)	AH 97 (24·3)	Hospital wound		
								Pelvic		
						VH 29 (7·2)	VH 34 (8·5)	AH 20 (19·6)	AH 6 (6)	< 0·05
								VH 6 (21)	VH 1 (3)	< 0·05
								Hospital total		
								AH 42 (41·2)	AH 16 (16)	< 0·01
								VH 10 (34)	VH 8 (24)	0·41
								Home wound		
								Pelvic		
								AH 9 (8·8)	AH 10 (10)	0·81
								VH 2 (7)	VH 1 (3)	VH 0·59
								Home total		
								AH 15 (14·7)	AH 25 (26)	AH 0·05
								VH 7 (24)	VH 10 (29)	VH 0·02
				Mezlocillin 5 g		AH 102 (25·5)	AH 101 (25·3)	Hospital wound		
								Pelvic		
						VH 29 (7·2)	VH 37 (9·2)	AH 20 (19·6)	AH 18 (17·8)	0·86
								VH 6 (21)	VH 0 (0)	< 0·01
								Hospital total		
								AH 42 (41·2)	AH 30 (29·7)	0·11
								VH 10 (34)	VH 6 (16)	0·15
								Home wound		
								Pelvic		
								AH 9 (8·8)	AH 4 (4·0)	0·25
								VH 2 (7)	VH 0 (0)	0·19
								Home total		
								AH 15 (14·7)	AH 14 (13·9)	1·000
								VH 7 (24)	VH 2 (5)	0·04
Dhadwal *et al*.^38^	CABG	NNIS infection risk score^35^ CDC sternal wound^50^	Cefuroxime 1·5 g	Rifampicin 600 mg; gentamicin 2 mg/kg; vancomycin 15 mg/kg	201 (165 : 36)	106 (52·8)	95 (47·2)	NNIS 30‐day infection		
								12 (11·3)	4 (4)	0·063
								Sternal wound (90 days)		
								25 (23·6)	8 (8)	0·004¶
								Superficial		
								11 (10·4)	4 (4)	0·097
								Deep		
								8 (7·5)	2 (2)	0·15#
								Organ space		
								6 (5·7)	2 (2)	0·36#
								Deep + organ space		
								14 (13·2)	4 (4)	0·03
								Sternal debridement		
								19 (17·9)	4 (4)	0·002
								Harvest site infection		
								7 (6·6)	45 (5)	0·69
Dijksman *et al*.^39^	Digestive tract surgery	Wound infection, intra‐abdominal abscess and anastomotic leak^47,51^. Calculated event rate was percentage of patients who suffered at least 1 infectious complication	Placebo. Parenteral perioperative antibiotic cefuroxime 1500 mg + metronidazole 500 mg	SDD (polymyxin B sulphate100 mg + tobramycin 80 mg + amphotericin B 500 mg). Parenteral perioperative antibiotic cefuroxime 1500 mg + metronidazole 500 mg	289 (156 : 133)	146 (50·5)	143 (49·5)	45 (30·8)	28 (19·6)	0·03¶
Garcia‐Rodriguez *et al*.^40^ **	Gastroduodenal or biliary surgery	Surgical wound infection: cellulitis with purulent secretion, with or without dehiscence (NRC^52^)	Cefoxitin 2 g	Cefotaxime 1 g	1451 (624 : 827)	716 (50·2)	722 (49·8)	Wound infection		
								54 (7·5)	24 (3·3)	< 0·002
Jones *et al*.^41^ ††	Gastrointestinal; gynaecological, orthopaedic (total joint replacement and open reduction of fractures) and other surgery	Postoperative surgical incision or peritoneal cavity infection	Cefotaxime 1 g	Cefoperazone 1 g	812 (42 : 770)	401 (49·4)	411 (50·6)	Wound infection		
								12 (3·0)	9 (2·2)	> 0·05
					Total general	96	89	1 (1)	2 (2)	1·000
					UGIT	72	66	0 (0)	0 (0)	
					Colorectal	24	23	1 (4)	2 (9)	1·000
					Total O+G	168	168	9 (5·4)	6 (3·6)	0·60
					Hysterectomy	119	125	8 (6·7)	6 (4·8)	0·59
					C‐section	19	18	1 (5)	0 (0)	1·000
					Other O+G	30	25	0 (0)	0 (0)	
					Total orthopaedic	74	77	1 (1)	0 (0)	0·49
					Total joints	51	59	0 (0)	0 (0)	
					Other orthopaedic	23	18	1 (4)	0 (0)	1·000
					Other surgery	61	77	1 (2)	1 (1)	1·000
Marroni *et al*.^42^ ‡‡	Abdominal aortic or lower limb prosthetic vascular surgery	Surgical wound infection; deep wound infection (CDC^53^)	Cefazolin 2 g	Teicoplanin 400 mg	238 (220 : 18)	119 (50·0)	119 (50·0)	SSI		
								2 (1·7)	7 (5·9)	0·19
								Graft		
								2 (1·7)	0 (0·0)	0·49
								Wound		
								5 (4·2)	2 (1·7)	0·46
Matkaris *et al*.^43^	AH	Fever > 38°C for 24 h, blood analysis, urine analysis, clinical evaluation	No prophylaxis	Ceftriaxone 2 g	200 (0 : 200)	50 (25·0)	50 (25·0)	15 (30)	3 (6)	< 0·01§§
				Cefotaxime 2 g			50 (25·0)		4 (8)	
				Ceftazidime 2 g			50 (25·0)		4 (8)	
Matsui *et al*.^44^ ¶¶	Laparoscopic cholecystectomy for removal of gallbladder stones or polyps	SSI (surgical wound and subhepatic abscess)	No prophylaxis	Cefazolin 1 g	1037 (490 : 547)	519 (50·0)	518 (50·0)	SSI		
								19 (3·7)	4 (0·8)	0·001
								Wound		
								16 (3·1)	4 (0·8)	0·005
								Subhepatic		
								3 (0·6)	0 (0·0)	0·249
								All infections		
								35 (6·7)	6 (1·2)	< 0·001
Sisto *et al*.^45^ ##	CABG	Superficial and deep sternal wound infection; donor‐site infection	Ceftriaxone 2 g	Cefuroxime 1·5 g, then cefuroxime 1·5 g 8‐hourly until end of day 2 after surgery	551 (437 : 114)	274 (49·7)	277 (50·3)	Superficial		
								4 (1·5)	7 (2·5)	0·56
								Deep		
								8 (2·9)	8 (2·9)	1·00
								Donor site		
								3 (1·1)	4 (1·4)	1·00
Wilson *et al*.^46^ ***	Colorectal surgery	SSI (organ space; deep incisional; either superficial infection or anastomotic leak) (NNIS^54,55^)	Ertapenem 1 g	Cefotetan 2 g	672 (365 : 307)	338 (50·3)	334 (49·7)	SSI		
								62 (18·3)	104 (31·1)	< 0·001
								Organ/space		
								4 (1·2)	12 (3·6)	0·05
								Deep		
								13 (3·8)	17 (5·1)	0·46
								Superficial		
								45 (13·3)	75 (22·5)	0·002
								Anastomotic leak		
								10 (3·0)	14 (4·2)	0·41

* Values in parentheses are percentages.

† Intervention failure results for cefazolin, clindamycin and cefoperazone were pooled as individual results were not stated; statistical method was not stated, but assumed to be Fisher's exact test.

‡ Fisher's exact test (*P* < 0·050 was considered significant with 80 per cent confidence level).

§ Analysis of significance in fourfold tables was done with the χ^2^ test with Yates' correction unless the total number of observations was less than 60 or the number in any cell was zero, when Fisher's exact test was used; threefold or greater tables were analysed with the χ^2^ test.

¶ χ^2^ or Fisher's exact test with two‐sided significance level of 0·05.

# χ^2^ test with Yates' correction.

** Intention‐to‐treat data; statistical analysis with Fisher's exact test; infection data were missing for six patients in the control group and seven in the intervention group.

†† Per‐protocol data; statistical analysis with Fisher's exact test or χ^2^ test; *P* < 0·050 considered significant;

‡‡ χ^2^ test with a two‐sided significance level of 0·05 when expected frequencies were less than 5.

§§ Statistical method not stated.

¶¶ χ^2^ test with significance level of 0·05; Fisher's exact test used for subhepatic comparison as expected frequencies in cells were less than 5.

## Student's *t* test for parametric data and Mann–Whitney or χ^2^ test for non‐parametric data; significance level of 0·05.

*** Per‐protocol data; absolute difference and 95 per cent c.i. for percentage prophylactic failure were determined in a statistical model adjusting for surgical procedure; 95 per cent c.i. that did not overlap zero indicated significant difference between groups at *P* < 0·050. n.s., Not stated; AH, abdominal hysterectomy; VH, vaginal hysterectomy; CABG, coronary artery bypass graft; NNIS, National Nosocomial Infections Surveillance; CDC, Centers for Disease Control and Prevention; SDD, selective decontamination of digestive tract; NRC, National Research Council; UGIT, upper gastrointestinal tract; O+G, obstetrics and gynaecology; C‐section, caesarean section; SSI, surgical‐site infection.

All studies reported SSI rates and the effectiveness of the preoperative antibiotic prophylaxis. Prophylactic effectiveness was demonstrated in 11 studies^35–44,46^, although effectiveness was statistically significant in only seven^37^, ^38^, ^39^, ^40^ ^43^, ^44^ ^46^. Blair and colleagues^35^ demonstrated effectiveness of the intervention compared with placebo, but failed to stipulate which of the three interventions was effective (cefazolin, clindamycin or cefoperazone). Effectiveness was therefore calculated for the pooled interventions. Matkaris *et al*.^43^ demonstrated significant effectiveness of three prophylactic antibiotics *versus* the no‐antibiotic control, and also reported comparable differences between the three prophylactic antibiotics. The study that did not demonstrate prophylactic effectiveness for the intervention compared a single dose of ceftriaxone (third‐generation cephalosporin) with three doses of cefuroxime (second generation) given three times daily, in patients undergoing CABG^45^.

Eleven studies^35^, ^36^, ^37^, ^38^, ^39^, ^40^, ^41^, ^42^, ^43^, ^44^ ^46^ reported length of hospital stay (LOS), although the reporting was inconsistent between treatment groups as well as between infected and non‐infected patients (*Table* 4). Overall LOS was reduced in the intervention group for all of the studies, although this was significant in only one study^44^. LOS was increased in the presence of infection compared with no infection in two studies^35^ ^40^. Five studies^38^, ^39^, ^40^ ^42^, ^45^ reported on mortality, although none stated the day of admission when the death occurred; there was no significant difference in mortality rates between intervention and control groups in the five studies^38^, ^39^, ^40^ ^42^, ^45^. There was one death from infection in each arm of the Marroni study^42^, whereas in the Sisto study^45^ no death was from infection. Mortality was not reported in the paper by Wilson *et al*.^46^, but was reported in the nested study of Itani and co‐workers^48^; the difference was not statistically significant and was not directly related to the prophylaxis.

**Table 4:** Length of hospital stay and mortality associated with preoperative prophylactic antibiotics

			Population		Length of hospital stay*					Mortality‡		
Reference	Surgical procedure	Preoperative prophylaxis	C	I	C	I	No infection	Infection	*P*	C	I	*P*
Blair *et al*.^35^ §	Neck dissection	No prophylaxis *versus* cefazolin, clindamycin and cefoperazone	99	93			8 (4–22)	23 (10–73)	n.c.	n.s.	n.s.	
Bold *et al*.^36^ ¶	Axillary lymph node dissection	Placebo (normal saline) *versus* cefonicid	90	88	5·9 (2–15)	3			n.c.	n.s.	n.s.	
Davey *et al*.^37^	AH or VH	AH: placebo (normal saline) *versus* cephradine	102	97	8·7 (8·2–9·2)	8·0 (7·7–8·3)			n.c	n.s.	n.s.	
		AH: placebo (normal saline) *versus* mezlocillin		101	8·7 (8·2–9·2)	7·9 (7·6–8·2)			n.c.	n.s.	n.s.	
		VH: placebo (normal saline) *versus* cephradine	29	34	7·2 (6·7–7·7)	8·1 (7·2–9·0)			n.c.	n.s.	n.s.	
		VH: placebo (normal saline) *versus* mezlocillin		37	7·2 (6·7–7·7)	7·3 (7·0–7·6)			n.c.	n.s.	n.s.	
Dhadwal *et al*.^38^ #	CABG	Cefuroxime *versus* rifampicin + gentamicin + vancomycin	106	95	11·7 (4–69)	9·5 (4–73)			0·063	4 (4)	1 (1)	0·630
Dijksman *et al*.^39^	Digestive tract surgery	Placebo, cefuroxime and metronidazole *versus* SDD, cefuroxime and metronidazole	146	143	(12, 9–18)	(11, 9–14)			0·055	5 (3·4)	6 (4·2)	0·732
Garcia‐Rodriguez *et al*.^40^ **	Gastroduodenal or biliary surgery	Cefoxitin *versus* cefotaxime	716	722	11·7 (4–69)	9·5 (4–73)	10·2 (9·9–10·5)	13·7 (12·4–15·0)	< 0·001	7 (0·6)	4 (0·6)	n.s.
Jones *et al*.^41^ ††	Hysterectomy, genitourinary, gastrointestinal and other (mainly orthopaedic total joint replacement and open reduction of fractures) surgery	Cefotaxime *versus* cefoperazone	401	411	11·5 (12–30)	14·3 (12–30)			n.c.	n.s.	n.s.	
Marroni *et al*.^42^	Abdominal aortic or lower limb prosthetic vascular surgery	Cefazolin *versus* teicoplanin	119	119	14·8	16·2			n.c.	3 (2·5)	4 (3·4)	1·000
Matkaris *et al*.^43^	AH	No antibiotic prophylaxis *versus* ceftriaxone	50	50	5·46	4·32			< 0·001	n.s.	n.s.	
		No antibiotic prophylaxis *versus* cefotaxime		50	5·46	4·36			< 0·001	n.s.	n.s.	
		No antibiotic prophylaxis *versus* ceftazidime		50	5·46	4·50			< 0·001	n.s.	n.s.	
Matsui *et al*.^44^ ‡‡	Laparoscopic cholecystectomy for removal of gallbladder stones or polyps	No antibiotic prophylaxis *versus* cefazolin	519	518	4·07(3·00)†	3·69(5·26)†			0·010	0 (0)	0 (0)	
Sisto *et al*.^45^	CABG	Ceftriaxone *versus* cefuroxime	274	277	n.s.	n.s.			n.c.	3 (1·1)	4 (1·4)	1·000
Wilson *et al*.^46^ §§	Colorectal surgery	Ertapenem *versus* cefotetan	338	334	7·6 (6·8–8·2)	8·7 (7·7–9·7)			n.c.	3 of 451 (0·7)	7 of 450 (1·6)	0·340

* Values are mean (median, range) unless indicated otherwise;

† values are mean(s.d.).

‡ Values in parentheses are percentages.

§ Infection rate and length of stay (LOS) for cefazolin, clindamycin and cefoperazone were pooled as individual results were not stated; mean cost per patient was based on length of hospital stay (LOS).

¶ Patients with infection were admitted to hospital (7 placebo, 1 intervention).

# Mann–Whitney *U* test for LOS and χ^2^ test with Yates' correction for mortality.

** Intention‐to‐treat data; infection data were missing for six patients in the control group and seven in the intervention group.

†† Per‐protocol data.

‡‡ Intention‐to‐treat data.

§§ Per‐protocol data; intention‐to‐treat data used for mortality reported in the nested study of Itani *et al*.^48^. C, control; I, intervention; n.c., not calculated (insufficient data in article); n.s., not stated; AH, abdominal hysterectomy; VH, vaginal hysterectomy; CABG, coronary artery bypass graft; SDD, selective decontamination of the digestive tract. *P* values are those reported in the article.

### Bacterial isolates and antimicrobial resistance

Six studies^38^ ^40^, ^41^, ^42^ ^45^, ^46^ reported and identified the bacterial pathogens responsible for SSIs; the pathogens were similar across the studies (*Table* 5). *Clostridium difficile*, a toxic organism found in the intestine causing colitis, was identified in one study^45^ after surgery following a second dose of cefuroxime. Wilson *et al*.^46^ also reported *C. difficile* colitis (in 2 patients who received ertapenem) and antimicrobial resistance of the pathogens to ertapenem *versus* cefotetan in the nested study^48^. Resistance of pathogens to ertapenem was much lower (16 per cent) than that to cefotetan (67 per cent). Only two other studies^38^ ^41^ reported antimicrobial resistance. Dhadwal and colleagues^38^ found no increase in vancomycin‐resistant *Enterococcus* or methicillin‐resistant *Staphylococcus aureus* (MRSA) in CABG, although Gram‐positive bacteria resistant to rifampicin were identified in both control (cefuroxime) and investigation (rifampicin, vancomycin and gentamicin) groups. Jones and co‐workers^41^ found few pathogens (8 per cent) resistant to cefoperazone and, although no pathogens were resistant to cefotaxime, 72 per cent were inhibited by cefotaxime in several surgical procedures.

**Table 5:** Evidence of preoperative prophylactic antibiotics in bacterial isolates and resistance patterns

		Preoperative prophylaxis		Bacterial isolates		
Reference	Population	Control	Intervention	Control	Intervention	Bacterial resistance patterns
Dhadwal *et al*.^38^ *	Median sternotomy for primary CABG of at least one thoracic artery and at least one of four defined risk factors: 201	Cefuroxime 1·5 g (single dose), then cefuroxime 750 mg at reversal of anticoagulation 8 and 16 h after surgery	Rifampicin 600 mg (single dose), then gentamicin 2 mg/kg + vancomycin 15 mg/kg on induction of anaesthesia. Postoperative vancomycin 7·5 mg/kg at 12, 24 and 36 h	19 of 99	7 of 87	No increase in vancomycin‐ resistant *Enterococcus* or MRSA
				GNB: 15	GNB: 7	
				GPB: 10	GPB: 4	
				Rifampicin‐resistant GPB: 4	Rifampicin‐ resistant GPB: 1	
				Vancomycin‐ resistant GPB: 0	Vancomycin‐ resistant GPB: 0	
				Anaerobic: 2	Anaerobic: 1	
				Yeast: 1	Yeast: 1	
Garcia‐Rodriguez *et al*.^40^ †	Gastroduodenal or biliary surgery with at least one of the 11 defined risk factors: 1451	Cefoxitin 2 g (single i.v. dose), then cefoxitin 2 g 6,12 and 18 h after surgery	Cefotaxime 1 g (single dose)	*Escherichia coli* and *Staphylococcus aureus* most common; frequency and study group not mentioned		Not stated
Jones *et al*.^41^	Hysterectomy, genitourinary, gastrointestinal or other (total joint replacement and open reduction of fractures) surgical procedures: 812	Cefotaxime 1·0 g (slow i.v. bolus after anaesthesia but 30 min before incision). Additional cefotaxime 1·0 g given during surgery if procedure duration 2 h or more. For bowel surgery, standard bowel preparation before prophylaxis	Cefoperazone 1·0 g (slow i.v. bolus after anaesthesia but 30 min before incision). For bowel surgery, standard bowel preparation before prophylaxis	12 of 21	18 of 21	Aerobic organisms 92% susceptible to cefoperazone and 72% inhibited by cefotaxime
				GNB: 2	GNB: 2	
				GPB: 5	GPB: 3	
				Anaerobic: 3	Anaerobic: 2	
Marroni *et al*.^42^	Abdominal aortic or lower limb prosthetic vascular surgery: 238	Cefazolin 2 g (single i.v. dose)	Teicoplanin 400 mg (single dose)	Graft		n.s.
				MRSA: 0	MRSA: 0	
				SWI		
				GNB: 1	GNB: 2	
				GPB: 1	GPB: 1	
				UTI		
				GNB: 3	GNB: 4	
				Bloodstream		
				GNB: 2	GNB: 0	
Sisto *et al*.^45^	CABG: 551	Ceftriaxone 2 g (single dose)	Cefuroxime 1·5 g (single dose), then cefuroxime 1·5 g 8‐hourly until end of postoperative day 2	Mediastinitis		n.s.
				GNB: 1	GNB: 1	
				GPB: 6	GPB: 4	
				Anaerobic: 0	Anaerobic: 1	
				*Clostridium difficile*: 0	*C. difficile*: 1	
Wilson *et al*.^46^ ‡	Colorectal surgery: 672)	Ertapenem 1 g (single dose)	Cefotetan 2 g (single dose)	GPB: 42	GPB: 51	67% resistant to cefotetan; 16% resistant to ertapenem
				Anaerobic: 36	Anaerobic: 44	
				GNB: 17	GNB: 23	
				*C. difficile*: 2		

* Intention‐to‐treat data for antibiotic efficacy.

† Infection data were missing for six patients in the control group and seven in the intervention group.

‡ Per‐protocol data; bacterial isolates and susceptibility data from nested study by Itani *et al*.^48^. GNB, Gram‐negative bacteria; GPB, Gram‐positive bacteria; MRSA, methicillin‐resistant *Staphylococcus aureus*; SWI, surgical wound infection; UTI, urinary tract infection; CABG, coronary artery bypass graft.

### Quality assessment of economic evaluation

A modified Drummond checklist^27^ was used to assess economic methodological quality for each study (*Table* 6; *Table S5*, supporting information). Overall four studies^39^, ^40^, ^41^ ^46^ were evaluated as being of high quality, six^36–38,43–45^ as moderate/acceptable quality, and two^35^ ^42^ as low/unacceptable quality. All studies defined an answerable question and included an alternative treatment. Eight studies^37–41,44–46^ accurately measured their outcomes and costs, which were both reported in the appropriate units. No study performed sensitivity analysis or discounted cost, although discounting was not applicable in six studies^37^ ^39^, ^41^ ^44^, ^45^, ^46^. Only one study^39^ performed an ICER analysis.

**Table 6:** Summary of quality assessment checklist for assessing economic evaluations of included studies

	No. of studies reporting (*n* = 12)			
Question	Yes	No	Unsure	Not applicable
Well defined question stated?	12	0	0	0
Description of alternatives?	12	0	0	0
Evidence of clinical effectiveness established?	10	1	1	0
Relevant costs and outcomes identified?	7	5	0	0
Costs measured accurately in appropriate units?	8	4	0	0
Outcomes measured accurately in appropriate units	8	4	0	0
Costs valued credibly?	10	2	0	0
Outcomes valued credibly?	10	2	0	0
Costs discounted? (*n* = 6)	0	6	0	6
Was incremental analysis performed?	1	11	0	0
Was sensitivity analysis performed?	1	11	0	0
Was generalizability discussed?	2	10	0	0

### Cost analysis of antibiotic prophylaxis

Of the included studies, nine^35^, ^36^, ^37^, ^38^ ^40^, ^42^ ^43^, ^45^ ^46^ were cost‐benefit studies, two were cost‐effectiveness studies^39^ ^44^ and one^41^ was a cost containment study (*Table* 1; *Table S3*, *supporting information*). These were all from the perspective of the healthcare provider, with costs reported as mean cost per patient or per patient episode. Sources for the cost data were reported in all studies, and costs included prophylactic antibiotic, daily hospital charge, nursing/staff time, hospital care, care after discharge, and treatment of the SSIs (*Table* 7). The currencies reported were: euros^39^, British pounds^37^, US dollars^35^ ^36^, ^38^ ^40^, ^41^, ^42^, ^43^, ^44^, ^45^, ^46^, drachma^43^ and pesetas^40^; both drachma and pesetas were converted to US dollars, which was the currency used in all cost analyses. Only four studies^39^ ^40^, ^42^ ^46^ reported the price year for the currency conversion. Nine studies^35,36,38–40,43–46^ reported cost savings favouring the use of the preoperative prophylaxis intervention and two^37^ ^42^ reported cost savings favouring the control prophylaxis. Davey and colleagues^37^ showed significant clinical effectiveness for cephradine and mezlocillin in abdominal and vaginal hysterectomy, but neither intervention was considered cost‐effective. One study^39^ reported an ICER when using selective decontamination of the digestive tract *versus* placebo in gastrointestinal surgery, with the prevention of at least one infection leading to a reported saving of €23 164 per patient. No study discounted costs, although Dijksman *et al*.^39^ stated that the reason for not discounting costs included a 1‐year time horizon, and they did perform a sensitivity analysis. One study^45^ considered only the acquisition and delivery cost of the antibiotic prophylaxis and not the treatment failures.

**Table 7:** Summary of reported costs and incremental cost‐effectiveness ratio calculated from study data

Reference	Intervention *versus* control	Intervention failure*	Control failure*	Treatment effect (TEc − TEi)	Mean cost of intervention (includes treatment cost)	Mean cost of control (includes treatment cost)	Incremental cost per patient	Incremental cost per patient (2016 €)†	ICER (2016 €)†
Blair *et al*.^35^ ‡	Cefazolin, clindamycin and cefoperazone *versus* placebo	3 of 93 (3)	10 of 99 (10)	7	$36 240·00	$36 030·00	$210·00	293·79	Dominant
Bold *et al*.^36^ §	Cefonicid *versus* placebo	5 of 88 (6)	12 of 90 (13)	7	$149·80	$364·87	−$215·07	−269·26	Dominant
Davey *et al*.^37^ ¶	AH: cephradine *versus* placebo	40 of 97 (41)	53 of 102 (52·0)	11	£18·26	£31·34	−£13·08	−37·92	Dominant
	AH: mezlocillin *versus* placebo	40 of 101 (39·6)	53 of 102 (52·0)	12·4	£17·61	£31·34	−£13·73	−37·92	Dominant
	VH: cephradine *versus* placebo	14 of 34 (41)	15 of 29 (52)	11	£40·60	£41·20	−£0·60	−1·65	Dominant
	VH: mezlocillin *versus* placebo	7 of 37 (19)	15 of 29 (52)	33	£8·80	£41·20	−£32·40	−89·50	Dominant
Dhadwal *et al*.^38^ #	Rifampicin +gentamicin +vancomycin *versus* cefuroxime	8 of 87 (9)	25 of 99 (25)	16	$15 158·00	$19 054·00	−$3896·00	−4315·99	Dominant
Dijksman *et al*.^39^ **	SDD (amphotericin B, polymyxin B sulphate + tobramycin) *versus* placebo	28 of 143 (19·6)	45 of 146 (30·8)	11·2	€12 031·00	€14 635·00	−€2604·00	−2731·28	Dominant
Garcia‐Rodriguez *et al*.^40^ ††	Cefotaxime *versus* cefoxitin	22 of 722 (3·3)	54 of 716 (7·7)	4·4	$28·64	$104·43	−$75·79	−120·72	Dominant
Jones *et al*.^41^ ‡‡	Cefoperazone *versus* cefotaxime	9 of 411 (2·2)	12 of 401 (3·0)	0·8	$14·50	$12·90	$1·60	2·64	5·12
Marroni *et al*.^42^ §§	Cefazolin *versus* teicoplanin	7 of 119 (5·9)	2 of 119 (1·7)	−4·2	$4803·13	$4361·86	$441·27	552·45	Dominated by control
Matkaris *et al*.^43^ ¶¶	Ceftriaxone *versus* no antibiotic	3 of 50 (6)	15 of 50 (30)	24	$150·12	$248·03	−$97·91	−140·10	Dominant
	Cefotaxime *versus* no antibiotic	4 of 50 (8)	15 of 50 (30)	22	$128·06	$248·03	−$119·97	−171·67	Dominant
	Ceftazidime *versus* no antibiotic	4 of 50 (8)	15 of 50 (30)	22	$137·81	$248·03	−$110·22	−157·71	Dominant
Matsui *et al*.^44^ ##	Cefazolin *versus* no antibiotic	6 of 518 (1·2)	35 of 519 (6·7)	5·5	$766·10	$831·90	−$65·80	−60·75	Dominant
Sisto *et al*.^45^ ***	Ceftriaxone *versus* cefuroxime	21 of 274 (7·7)	23 of 277 (8·3)	0·6	$36·11	$107·82	−$71·71	−95·95	Dominant
Wilson *et al*.^46^ †††	Ertapenen *versus* cefotetan	143 of 334 (42·8)	95 of 338 (28·1)	−14·7	$15 230·00	$17 411·00	−$2181·00	−2340·81	Dominant

* Values in parentheses are percentages.

† ‘Discounted’ cost per patient and incremental cost‐effectiveness ratio (ICER) calculated by means of a two‐step discounting process using the Campbell and Cochrane Economics Methods Group–Evidence for Policy and Practice Information and Coordinating Centre cost converter web‐based tool^32,33^. The 2016 implied conversion factor is US $1 = £0·70 sterling; the 2016 euro conversion factor is £1 sterling = €1·28.

‡ Treatment effects of cefazolin, clindamycin and cefoperazone were pooled, and costs were pooled and averaged; cost inferred from study setting to be US$; for conversion of 1992 US dollars to 2016 British pounds, the implied inflation factor for US $1 in 1992 to 2016 value is 1·57.

§ Price year inferred from publication date; for conversion of 1998 US dollars to 2016 British pounds, the implied inflation factor for US $1 in 1998 to 2016 is 1·41.

¶ Price year inferred from publication date; for conversion of 1988 British pounds to 2016 British pounds, the implied inflation factor for £1 sterling in 1988 to 2016 is 2·16.

# Price year inferred from study end date; cost data based on per‐protocol analysis; for conversion of 2004 US dollars to 2016 British pounds, the implied inflation factor for US $1 in 2004 to 2016 is 1·24.

** For conversion of 2008 euros to 2016 euros, the implied inflation factor for €1 in 2008 to 2016 is 1·05.

†† Cost inferred from study setting to be US$; for conversion of 1988 US dollars to 2016 British pounds, the implied inflation factor for US $1 in 1988 to 2016 is 1·79; infection data were missing for six patients in the control group and seven in the intervention group.

‡‡ Price year inferred from publication date; all treatment failures; for conversion of 1987 US dollars to 2016 British pounds, the implied inflation factor for US $1 in 1987 to 2016 is 1·87.

§§ Price year inferred from study end date; for conversion of 1998 US dollars to 2016 British pounds, the implied inflation factor for US $1 in 1998 to 2016 is 1·41.

¶¶ Price year inferred from publication date; for conversion of 1991 US dollars to 2016 British pounds, the implied inflation factor for US $1 in 1991 to 2016 is 1·61.

## Price year inferred from publication date; for conversion of 2013 US dollars to 2016 British pounds, the implied inflation factor for US $1 in 2013 to 2016 is 1·04.

*** Price year inferred from study end date; for conversion of 1994 US dollars to 2016 British pounds, the implied inflation factor for US $1 in 1994 to 2016 is 1·50.

††† Cost inferred from study setting to be US$; cost data based on per‐protocol analysis; for conversion of 2005 US dollars to 2016 British pounds, the implied inflation factor for US $1 in 2005 to 2016 is 1·21. TEc, treatment effect for control; TEi, treatment effect for intervention; AH, abdominal hysterectomy; VH, vaginal hysterectomy; SDD, selective decontamination of digestive tract. A more detailed version of this table is available as *Table S6*, supporting information.

### Calculated incremental cost‐effectiveness ratio

The calculated ICER was based on the results of each study, their reported currency and euros (2016) (*Table* 7; *Table S6, supporting information*). Eight studies did not clearly state the price year for the cost calculations, so the year in which the study ended^38^ ^42^, ^45^ and date of publication^36^ ^37^, ^41^ ^43^, ^44^ were used. The calculated treatment effect showing the proportion of infections averted ranged from 0·06 per cent in clean CABG surgery^45^ to 0·33 per cent in clean‐contaminated vaginal hysterectomy^37^, with one study^42^ showing a negative effect in vascular prosthetic surgery. The intervention in ten studies^35–40,43–46^ was dominant (more effective and cheaper than the control) and in one study^42^ the intervention was dominated by the control (it was less effective and more expensive). In the remaining study^41^, the intervention was more effective and more expensive than the control. This resulted in an incremental increase of €2·64 per patient and a resultant ICER of €5·12 for the year 2016.

## Discussion

This review aimed to evaluate the cost‐effectiveness of preoperative antibiotic prophylaxis in preventing SSIs, including assessment of the reporting quality of the clinical and cost‐effectiveness. Twelve studies published between 1988 and 2014 were identified, and included preoperative antibiotic prophylaxis as well as costs. Most of the studies had a large sample size: five had more than 500 participants, four had between 200 and 500 participants and three had fewer than 200 participants. All studies reported some measure of costs, but only two reported on incremental cost‐effectiveness and none included any of the recommended economic checklists^27^ ^31^. All identified studies reported on prophylactic effectiveness, although few included antibiotic resistance and none addressed the appropriateness of antibiotic stewardship.

Prophylactic effectiveness was achieved in ten studies. The size of these effects is considered clinically important, particularly in contaminated and clean‐contaminated surgery^37^ ^39^, ^40^, ^41^ ^44^, ^46^, which has a higher risk of baseline SSI compared with clean procedures^57^. Five^35^ ^36^, ^38^ ^42^, ^45^ of the included studies involved clean surgical procedures, so clinical effectiveness in four^35^ ^36^, ^38^ ^42^ of these studies was not unexpected. Prophylactic effectiveness was also achieved even when the comparator was another antibiotic^38^ ^40^, ^41^ ^46^. Most of the prophylactic interventions involved first‐, second‐ or third‐generation cephalosporins compared with either placebo or a control. Cephalosporins are safe and have a long half‐life, ensuring penetration of tissues^21^. They offer cover against most *S. aureus* strains and some Gram‐negative organisms, but not coagulase‐negative staphylococci or MRSA^22^. Only two studies mentioned screening for *C. difficile*. Cephalosporins, especially third‐generation drugs, have been linked to patients having an increased risk of colonization with *C. difficile*, causing toxic *C. difficile* colitis^22^, even when administered as a single dose^58^ ^59^. The size and dosage of antibiotic prophylaxis is important, as single‐dose administration may precipitate resistance unless the prophylactic drug has a sufficient half‐life and tissue penetration. One study showed that a single dose of the intervention (cefoperazone) was less effective clinically and cost more than control prophylaxis (cefotaxime). Both of these antibiotics are third‐generation cephalosporins, and both were administered as a single bolus 30 min after anaesthesia but before incision. Cefotaxime was administered again during surgery if the duration of the procedure exceeded 2 h.

Teicoplanin, a glycopeptide, may also be administered as a single dose. Its use as an intervention, however, was less effective and more expensive compared with cefazolin (a first‐generation cephalosporin). Cefazolin remains the prophylactic choice in vascular surgery as it is effective against *S. aureus* (the most frequently isolated organism in infected vascular wounds). Cefazolin has been shown to be as effective as cefamandole and cefuroxime in prosthetic vascular surgery^60^. With the increase in MRSA, vancomycin is an alternative, but it is toxic. Teicoplanin is similar to vancomycin, but is less toxic and has a longer half‐life, so may be administered once daily. Teicoplanin lacks activity against Gram‐negative bacteria, however, and most infections in the teicoplanin study were caused by Gram‐negative bacteria; this may have contributed to the increased costs per patient.

Combining the findings of economic evaluations with those of clinical‐effectiveness trials provides healthcare policy‐makers with evidence‐based options for healthcare decision‐making. The methodology of economic evaluations needs to be defined clearly at the study outset. This review identified low to acceptable reporting of the economic evaluations, but with great variation, whereas the reporting of clinical effectiveness was more standardized. The most recent studies were more consistent in terminology and reporting of costs and their units. Some of the studies did not include treatment failures in their cost analysis, and this may result in an intervention that is cost‐saving but not necessarily cost‐effective. In addition, cost‐effectiveness may be more favourable in procedures that carry a higher baseline risk of SSI when the cost of prophylaxis is the same. Length of hospital stay is a recognized factor contributing to costs^7^, ^8^, ^9^ ^11^, and all studies reported a reduced length of stay compared with the control regimen; however, it was difficult to determine the exact costs of the stay. It is also recognized that mean daily costs decrease with extended length of stay, with the most intensive costs incurred in the period shortly after admission^9^; this may be perceived as a disincentive for hospitals to eliminate all SSIs^9^ ^10^. None of the included studies reported decreasing costs of the hospital admission; all reported a daily hospital charge. Mortality also has an associated cost, and in cost‐effectiveness studies is considered a permanent sequela. Only five studies and a nested study reported mortality, and none included deaths in the cost analysis.

The methodological quality of the included studies was not well reported, as evidenced by low scores on the CHEERS checklist^31^, whereas economic reporting was moderate to high, with seven studies ranking 75 per cent or above on the modified Drummond quality checklist^27^. Two of the highest‐quality studies were among the most recent ones, published in 2008 and 2012. There was, however, no standard method of reporting costs, and some cost components were not always reported; discounting was not reported in any study. Consistent inclusion of standardized economic studies in clinical trials and quasi‐experimental studies would allow evidence‐based decision‐making with respect to antibiotic efficacy and cost‐effectiveness.

This review has five main limitations. First, the search terms used may not have identified all articles, as a wide variety of terms exist to describe economic evaluations, prophylaxis and infection. Second, the review was restricted to studies performed in OECD countries. The purpose of the restriction was to reduce the effect of differences in operating theatre conditions and surgical procedures on the incidence of SSI. Third, the ICER analysis is based on the published study data and, because there was heterogeneity between the studies and sensitivity analysis was not always reported, it was limited to point estimates. Fourth, in this review, an ICER was not sensitive enough to rank cost‐effectiveness, as most of the interventions were dominant. For the dominant interventions using an ICER the range of difference could not be determined, and possibly a quality‐adjusted life‐year framework would be more suitable; however, this would require standardized reporting. Fifth, despite the importance of preventing primary antibiotic resistance, the review did not attempt to address the development of resistance or antibiotic stewardship, because no study reported on either. This also implies that the results of these studies have limited generalizability; if resistance patterns differ, a drug that is (cost‐)effective in one context may not be in another. The specific findings of the studies reviewed here should therefore be treated with caution.

The strengths of this review are several. It is the first to include both clinical and economic effectiveness of preoperative prophylaxis; it included five databases, and the numerous keywords were matched with indexed terms specific to the databases. This review summarized large data sets that encompassed many surgical specialties and procedures. It is recommended^29^ ^30^, ^61^ that more than one reviewer should screen for papers to be included in a systematic review. This review used two independent reviewers, and the κ statistic for each level of screening was at the higher end of the scale (from substantial to almost perfect).

This review of the cost‐effectiveness of preoperative antibiotic prophylaxis found that most interventions were cost‐effective. To ensure that preoperative prophylaxis continues to prevent SSI, there needs to be increased awareness of the prevalence of resistance within each facility and improved antibiotic stewardship to reduce the development of resistance. Antibiotic stewardship includes use of the appropriate recommended antibiotic prophylaxis based on the most common pathogens likely to cause SSI for a specific surgical procedure, following recommended timing of administration before incision to ensure maximum tissue concentration, adjusting the prophylaxis dose according to the patient's bodyweight, redosing the prophylaxis at intervals of two half‐lives, and discontinuing prophylaxis after surgery within recommended time frames. New antibiotic prophylaxis regimens may be implemented when they are less effective or more expensive if economic methods are not included routinely in RCTs and quasi‐experimental studies. Economic methods would improve the understanding and true economic benefit of these new regimens. The economic methods need to be standardized against recommended guidelines and incorporate sensitivity analysis, discount rates, year and date of the study, unit costs, mortality, treatment effects, antibiotic resistance and quality‐of‐life costs.

## Disclosure

The authors declare no conflict of interest.

## Supporting information


**Appendix S1**
**Table S1** Database search terms including complete searches for Cumulative Index to Nursing and Allied Health Literature (CINAHL) and Web of Science (WOS)
**Table S2** List of OECD countries*
**Table S3** CHEERS checklist of reporting quality
**Table S4** Quality assessment checklist for assessing economic evaluations of included studies

## References

[1] de Lissovoy G , Fraeman K , Hutchins V , Murphy D , Song D , Vaughn BB . Surgical site infection: incidence and impact on hospital utilization and treatment costs. Am J Infect Control 2009; 37: 387–397. 19398246 10.1016/j.ajic.2008.12.010

[2] Anderson DJ . Surgical site infections. Infect Dis Clin North Am 2011; 25: 135–153. 21315998 10.1016/j.idc.2010.11.004

[3] Ozdemir S , Gulpinar K , Ozis SE , Sahli Z , Kesikli SA , Korkmaz A *et al* The effects of preoperative oral antibiotic use on the development of surgical site infection after elective colorectal resections: a retrospective cohort analysis in consecutively operated 90 patients. Int J Surg 2016; 33: 102–108. 27463886 10.1016/j.ijsu.2016.07.060

[4] Horan TC , Gaynes RP , Martone WJ , Jarvis WR , Emori TG . CDC definitions of nosocomial surgical site infections, 1992: a modification of CDC definitions of surgical wound infections. Infect Control Hosp Epidemiol 1992; 13: 606–608. 1334988

[5] Lankiewicz JD , Yokoe DS , Olsen MA , Onufrak F , Fraser VJ , Stevenson K *et al* Beyond 30 days: does limiting the duration of surgical site infection follow‐up limit detection? Infect Control Hosp Epidemiol 2012; 33: 202–204. 22227993 10.1086/663715 PMC3608264

[6] Boltz MM , Hollenbeak CS , Julian KG , Ortenzi G , Dillon PW . Hospital costs associated with surgical site infections in general and vascular surgery patients. Surgery 2011; 150: 934–942. 21676424 10.1016/j.surg.2011.04.006

[7] Dimick JB , Chen SL , Taheri PA , Henderson WG , Khuri SF , Campbell DA . Hospital costs associated with surgical complications: a report from the private‐sector National Surgical Quality Improvement Program. J Am Coll Surg 2004; 199: 531–537. 15454134 10.1016/j.jamcollsurg.2004.05.276

[8] Broex EC , van Asselt AD , Bruggeman CA , van Tiel FH . Surgical site infections: how high are the costs? J Hosp Infect 2009; 72: 193–201. 19482375 10.1016/j.jhin.2009.03.020

[9] Shepard J , Ward W , Milstone A , Carlson T , Frederick J , Hadhazy E *et al* Financial impact of surgical site infections on hospitals: the hospital management perspective. JAMA Surg 2013; 148: 907–914. 23965750 10.1001/jamasurg.2013.2246

[10] Jenks PJ , Laurent M , McQuarry S , Watkins R . Clinical and economic burden of surgical site infection (SSI) and predicted financial consequences of elimination of SSI from an English hospital. J Hosp Infect 2014; 86: 24–33. 24268456 10.1016/j.jhin.2013.09.012

[11] Plowman R , Graves N , Griffin MA , Roberts JA , Swan AV , Cookson B *et al* The rate and cost of hospital‐acquired infections occurring in patients admitted to selected specialties of a district general hospital in England and the national burden imposed. J Hosp Infect 2001; 47: 198–209. 11247680 10.1053/jhin.2000.0881

[12] Leaper DJ , Van Goor H , Reilly J , Petrosillo N , Geiss HK , Torres AJ *et al* Surgical site infection – a European perspective of incidence and economic burden. Int Wound J 2004; 1: 247–273. 16722874 10.1111/j.1742-4801.2004.00067.x PMC7951634

[13] Schweizer ML , Cullen JJ , Perencevich EN , Vaughan Sarrazin MS . Costs associated with surgical site infections in Veterans Affairs hospitals. JAMA Surg 2014; 149: 575–581. 24848779 10.1001/jamasurg.2013.4663 PMC7393605

[14] Lee I , Agarwal RK , Lee BY , Fishman NO , Umscheid CA . Systematic review and cost analysis comparing use of chlorhexidine with use of iodine for preoperative skin antisepsis to prevent surgical site infection. Infect Control Hosp Epidemiol 2010; 31: 1219–1229. 20969449 10.1086/657134 PMC3833867

[15] Noorani A , Rabey N , Walsh SR , Davies RJ . Systematic review and meta‐analysis of preoperative antisepsis with chlorhexidine *versus* povidone–iodine in clean‐contaminated surgery. Br J Surg 2010; 97: 1614–1620. 20878942 10.1002/bjs.7214

[16] Gillespie BM , Chaboyer W , Erichsen‐Andersson A , Hettiarachchi RM , Kularatna S . Economic case for intraoperative interventions to prevent surgical‐site infection. Br J Surg 2017; 104: e55–e64. 10.1002/bjs.10428 28121042

[17] Gheorghe A , Roberts TE , Pinkney TD , Bartlett DC , Morton D , Calvert M . The cost‐effectiveness of wound‐edge protection devices compared to standard care in reducing surgical site infection after laparotomy: an economic evaluation alongside the ROSSINI trial. PLoS One 2014; 9: e95595. 10.1371/journal.pone.0095595 PMC3991705 24748154

[18] Patel A , Bergman A , Moore B , Haglund U . The economic burden of complications occurring in major surgical procedures: a systematic review. Appl Health Econ Health Policy 2013; 11: 577–592. 24166193 10.1007/s40258-013-0060-y

[19] Kao LS , Meeks D , Moyer VA , Lally KP . Peri‐operative glycaemic control regimens for preventing surgical site infections in adults. Cochrane Database Syst Rev 2009; (3)CD006806. 10.1002/14651858.CD006806.pub2 PMC2893384 19588404

[20] Beltramini AM , Salata RA , Ray AJ . Thermoregulation and risk of surgical site infection. Infect Control Hosp Epidemiol 2011; 32: 603–610. 21558774 10.1086/660017

[21] Bratzler DW , Dellinger EP , Olsen KM , Perl TM , Auwaerter PG , Bolon MK *et al*; American Society of Health-System Pharmacists; Infectious Disease Society of America; Surgical Infection Society; Society for Healthcare Epidemiology of America. Clinical practice guidelines for antimicrobial prophylaxis in surgery. Am J Health Syst Pharm 2013; 70: 195–283. 23327981 10.2146/ajhp120568

[22] Scottish Intercollegiate Guidelines Network (SIGN) . *Antibiotic Prophylaxis in Surgery*; SIGN Publication number 104; July 2008 [updated April 2014]. http://www.sign.ac.uk/assets/sign104.pdf%20%5baccessed%2016%20June%202017%5d].

[23] Anderson DJ , Podgorny K , Berrios‐Torres SI , Bratzler DW , Dellinger EP , Greene L *et al* Strategies to prevent surgical site infections in acute care hospitals: 2014 update. Infect Control Hosp Epidemiol 2014; 35: 605–627. 24799638 10.1086/676022 PMC4267723

[24] Higgins JPT , Green S (eds). Cochrane Handbook for Systematic Reviews of Interventions, Version 5.1.0; updated March 2011. http://handbook.cochrane.org [accessed 30 May 2014].

[25] Centre for Reviews and Dissemination (CRD), University of York . Systematic Reviews: CRD's Guidance for Undertaking Reviews in Health Care; 2009. https://www.crd.york.ac.uk/CRDWeb/GuideToSearching.asp [accessed 30 May 2014].

[26] Moher D , Liberati A , Tetzlaff J , Altman DG ; PRISMA Group . Preferred reporting items for systematic reviews and meta‐analyses: the PRISMA statement. BMJ 2009; 151: 264–269. PMC3090117 21603045

[27] Drummond MF , Sculpher MJ , Torrance GW , O'Brien BJ , Stoddart GL . Methods for the Economic Evaluation of Health Care Programmes (3rd ed.). Oxford University Press: New York, 2005.

[28] World Bank Group . *Data: Country and Lending Groups 2014* http://data.worldbank.org/about/country-and-lending-groups%20%5baccessed%2016%20July%202014%5d].

[29] Watson PF , Petrie A . Method agreement analysis: a review of correct methodology. Theriogenology 2010; 73: 1167–1179. 20138353 10.1016/j.theriogenology.2010.01.003

[30] Landis JR , Koch GG . The measurement of observer agreement for categorical data. Biometrics 1977; 33: 159–174. 843571

[31] Husereau D , Drummond M , Petrou S , Carswell C , Moher D , Greenberg D *et al*; CHEERS Task Force. Consolidated Health Economic Evaluation Reporting Standards (CHEERS) statement. BMJ 2013; 346: f1049. 10.1136/bmj.f1049 23529982

[32] Campbell and Cochrane Economics Methods Group (CCEMG) and Evidence for Policy and Practice Information and Coordinating Centre (EPPI‐Centre) . *CCEMG – EPPI‐Center Cost Converter,* v.1.5; April 2016. http://eppi.ioe.ac.uk/costconversion/default.aspx [accessed 20 May 2017].

[33] Shemilt I , Thomas J , Morciano M . A web‐based tool for adjusting costs to a specific target currency and price year. Evid Policy 2010; 6: 51–59.

[34] International Monetary Fund . *World Economic Outlook Database*; April 2016. https://www.imf.org/external/pubs/ft/weo/2016/01/weodata/index.aspx [accessed 20 May 2017].

[35] Blair EA , Johnson JT , Wagner RL , Carrau RL , Bizakis JG . Cost analysis of antibiotic prophylaxis in clean head and neck surgery. Arch Otolaryngol Head Neck Surg 1995; 121: 269–271. 7873141 10.1001/archotol.1995.01890030011002

[36] Bold RJ , Mansfield PF , Berger DH , Pollock RE , Singletary SE , Ames FC *et al* Prospective, randomized, double‐blind study of prophylactic antibiotics in axillary lymph node dissection. Am J Surg 1998; 176: 239–243. 9776150 10.1016/s0002-9610(98)00154-8

[37] Davey PG , Duncan ID , Edward D , Scott AC . Cost‐benefit analysis of cephradine and mezlocillin prophylaxis for abdominal and vaginal hysterectomy. Br J Obstet Gynaecol 1988; 95: 1170–1177. 3145014 10.1111/j.1471-0528.1988.tb06796.x

[38] Dhadwal K , Al‐Ruzzeh S , Athanasiou T , Choudhury M , Tekkis P , Vuddamalay P *et al* Comparison of clinical and economic outcomes of two antibiotic prophylaxis regimens for sternal wound infection in high‐risk patients following coronary artery bypass grafting surgery: a prospective randomised double‐blind controlled trial. Heart 2007; 93: 1126–1133. 17309908 10.1136/hrt.2006.103002 PMC1955036

[39] Dijksman LM , Roos D , Gerhards MF , Tijssen JG , Gouma DJ , Dijkgraaf MG . Cost‐effectiveness of perioperative selective decontamination of the digestive tract *versus* placebo in elective gastrointestinal surgery. Dig Surg 2012; 29: 384–390. 23128405 10.1159/000343095

[40] Garcia‐Rodriguez JA , Puig‐LaCalle J , Arnau C , Porta M , Vallve C . Antibiotic prophylaxis with cefotaxime in gastroduodenal and biliary surgery. Am J Surg 1989; 158: 428–432. 2510530 10.1016/0002-9610(89)90278-x

[41] Jones RN , Wojeski WV . Single‐dose cephalosporin prophylaxis of 929 surgical procedures in a prepaid group practice: a prospective, randomized comparison of cefoperazone and cefotaxime. Diagn Microbiol Infect Dis 1987; 6: 323–334. 3581737 10.1016/0732-8893(87)90183-0

[42] Marroni M , Cao P , Fiorio M , Maghini M , Lenti M , Repetto A *et al* Prospective, randomized, double‐blind trial comparing teicoplanin and cefazolin as antibiotic prophylaxis in prosthetic vascular surgery. Eur J Clin Microbiol Infect Dis 1999; 18: 175–178. 10357049 10.1007/s100960050253

[43] Matkaris M , Markantes K , Stayannis K , Iatrakis G , Kourounis G , Tzingounis V . Reduction of hospital cost and administration of prophylactic antibiotherapy in gynecological surgery. Isr J Med Sci 1991; 27: 134–136. 2016152

[44] Matsui Y , Satoi S , Kaibori M , Toyokawa H , Yanagimoto H , Matsui K *et al* Antibiotic prophylaxis in laparoscopic cholecystectomy: a randomized controlled trial. PLoS One 2014; 9: e106702. 10.1371/journal.pone.0106702 PMC4156368 25192389

[45] Sisto T , Laurikka J , Tarkka MR . Ceftriaxone *vs* cefuroxime for infection prophylaxis in coronary bypass surgery. Scand J Thorac Cardiovasc Surg 1994; 28: 143–148. 7792559 10.3109/14017439409099119

[46] Wilson SE , Turpin RS , Kumar RN , Itani KM , Jensen EH , Pellissier JM *et al* Comparative costs of ertapenem and cefotetan as prophylaxis for elective colorectal surgery. Surg Infect (Larchmt) 2008; 9: 349–356. 18570576 10.1089/sur.2007.047

[47] Roos D , Dijksman LM , Oudemans‐van Straaten HM , de Wit LT , Gouma DJ , Gerhards MF . Randomized clinical trial of perioperative selective decontamination of the digestive tract *versus* placebo in elective gastrointestinal surgery. Br J Surg 2011; 98: 1365–1372. 21751181 10.1002/bjs.7631

[48] Itani KMF , Wilson SE , Awad SS , Jensen EH , Finn TS , Abramson MA . Ertapenem *versus* cefotetan prophylaxis in elective colorectal surgery. N Engl J Med 2006; 355: 2640–2651. 17182989 10.1056/NEJMoa054408

[49] Johnson JT , Myers EN , Thearle PB , Sigler BA , Schramm VL Jr. Antimicrobial prophylaxis for contaminated head and neck surgery. Laryngoscope 1984; 94: 46–51. 6361430 10.1002/lary.5540940111

[50] Jonkers D , Elenbaas T , Terporten P , Nieman F , Stobberingh E . Prevalence of 90‐days postoperative wound infections after cardiac surgery. Eur J Cardiothorac Surg 2003; 23: 97–102. 12493512 10.1016/s1010-7940(02)00662-0

[51] Rommes JH , Rios G , Zandstra DF . Therapy of infection. Trends Anaesth Crit Care 2001; 12: 25–33.

[52] Berard F , Gandon J . Postoperative wound infections: the influence of ultraviolet irradiation of the operating room and of various other factors. Ann Surg 1964; 160(Suppl 2): 1–192. 14179433

[53] Garner JS , Jarvis WR , Emori TG , Horan TC , Hughes JM . CDC definitions for nosocomial infections, 1988. Am J Infect Control 1988; 16: 128–140. 2841893 10.1016/0196-6553(88)90053-3

[54] Horan TC , Culver DH , Gaynes RP , Jarvis WR , Edwards JR , Reid CR . Nosocomial infections in surgical patients in the United States, January 1986–June 1992. National Nosocomial Infections Surveillance (NNIS) System. Infect Control Hosp Epidemiol 1993; 14: 73–80. 8440883 10.1086/646686

[55] Mangram AJ , Horan TC , Pearson ML , Silver LC , Jarvis WR . Guideline for prevention of surgical site infection, 1999. Hospital Infection Control Practices Advisory Committee. Infect Control Hosp Epidemiol 1999; 20: 250–278. 10219875 10.1086/501620

[56] Jarvis WR . Benchmarking for prevention: the Centers for Disease Control and Prevention's National Nosocomial Infections Surveillance (NNIS) system experience. Infection 2003; 31 **(** Suppl 2): 44–48. 15018472

[57] Bowater RJ , Stirling SA , Lilford RJ . Is antibiotic prophylaxis in surgery a generally effective intervention? Testing a generic hypothesis over a set of meta‐analyses. Ann Surg 2009; 249: 551–556. 19300236 10.1097/SLA.0b013e318199f202

[58] Privitera G , Scarpellini P , Ortisi G , Nicastro G , Nicolin R , de Lalla F . Prospective study of *Clostridium difficile* intestinal colonization and disease following single‐dose antibiotic prophylaxis in surgery. Antimicrob Agents Chemother 1991; 35: 208–210. 2014978 10.1128/aac.35.1.208 PMC244972

[59] Ambrose NS , Johnson M , Burdon DW , Keighley MR . The influence of single dose intravenous antibiotics on faecal flora and emergence of *Clostridium difficile* . J Antimicrob Chemother 1985; 15: 319–326. 3846592 10.1093/jac/15.3.319

[60] Edwards WH Jr, Kaiser AB , Tapper S , Edwards WH Sr, Martin RS III , Mulherin JL Jr *et al* Cefamandole *versus* cefazolin in vascular surgical wound infection prophylaxis: cost‐effectiveness and risk factors. J Vasc Surg 1993; 18: 470–476. 8377241 10.1067/mva.1993.48123

[61] Edwards P , Clarke M , DiGuiseppi C , Pratap S , Roberts I , Wentz R . Identification of randomized controlled trials in systematic reviews: accuracy and reliability of screening records. Stat Med 2002; 21: 1635–1640. 12111924 10.1002/sim.1190

