# Supplementary material for: Systematic review of the cost‐effectiveness of preoperative antibiotic prophylaxis in reducing surgical‐site infection
Source: BJS Open. 2018 Apr 14;2(3):81–98. doi: 10.1002/bjs5.45 (PMC5989978; doi:10.1002/bjs5.45)
Supplement: Supplementary file 1 — Appendix S1 Table S1 Database search terms including complete searches for Cumulative Index to Nursing and Allied Health Literature (CINAHL) and Web of Science (WOS) Table S2 List of OECD countries* Table S3 CHEERS checklist of reporting quality Table S4 Quality assessment checklist for assessing economic evaluations of included studies [file BJS5-2-81-s001.docx]

**BJS5_45**

**Systematic review of the cost-effectiveness of preoperative antibiotic prophylaxis in reducing surgical-site infection**

**J. Allen, M. David and J. L. Veerman**

**Table S1** Database search terms including complete searches for Cumulative Index to Nursing and Allied Health Literature (CINAHL) and Web of Science (WOS)

| Database;  Years searched | Database full name | Search | Search terms | Articles identified |
| --- | --- | --- | --- | --- |
| PubMed;  1970 to 2017 | PubMed Complementary and alternative medicine |  | ((("Surgical Wound Infection"[Mesh] OR "surgical site infection*"[All Fields] OR "surgical wound infection*"[All Fields] OR "postoperative wound infection*"[All Fields] " OR "Surgical infection" OR "Nosocomial infection" OR "Hospital infection" OR "hospital acquired infection" OR "post operative complication" OR "wound care"))) AND (prevent[All Fields] OR prevent'[All Fields] OR prevent'ivnom[All Fields] OR prevent4827[All Fields] OR preventa[All Fields] OR preventa'[All Fields] OR preventabile[All Fields] OR preventabilita[All Fields] OR preventabilities[All Fields] OR preventability[All Fields] OR preventability'[All Fields] OR preventabilni[All Fields] OR preventable[All Fields] OR preventable'[All Fields] OR preventables[All Fields] OR preventably[All Fields] OR preventaf[All Fields] OR preventage[All Fields] OR prevental[All Fields] OR preventan[All Fields] OR preventarthritis[All Fields] OR preventary[All Fields] OR preventation[All Fields] OR preventatitve[All Fields] OR preventative[All Fields] OR preventative'[All Fields] OR preventatively[All Fields] OR preventatives[All Fields] OR preventav[All Fields] OR preventavna[All Fields] OR preventavnej[All Fields] OR preventavno[All Fields] OR preventbeta[All Fields] OR preventcd[All Fields] OR preventchildabuse[All Fields] OR preventchildabusenc[All Fields] OR preventd[All Fields] OR prevente[All Fields] OR preventec[All Fields] OR prevented[All Fields] OR prevented'[All Fields] OR preventede[All Fields] OR preventely[All Fields] OR preventenance[All Fields] OR preventer[All Fields] OR preventer'[All Fields] OR preventers[All Fields] OR preventers'[All Fields] OR preventes[All Fields] OR preventesabeta[All Fields] OR preventi[All Fields] OR preventi'oban[All Fields] OR preventi'oj'anak[All Fields] OR preventia[All Fields] OR preventian[All Fields] OR preventibility[All Fields] OR preventible[All Fields] OR preventic[All Fields] OR preventica[All Fields] OR preventicare[All Fields] OR preventicare's[All Fields] OR preventice[All Fields] OR preventicniho[All Fields] OR preventico[All Fields] OR preventicon[All Fields] OR preventics[All Fields] OR preventicular[All Fields] OR preventicum[All Fields] OR preventid[All Fields] OR preventie[All Fields] OR preventiebeleid[All Fields] OR preventieconsult[All Fields] OR preventieconsult'[All Fields] OR preventiecursus[All Fields] OR preventieen[All Fields] OR preventief[All Fields] OR preventiefond[All Fields] OR preventiefonderzoek[All Fields] OR preventiefonds[All Fields] OR preventiegids[All Fields] OR preventiei[All Fields] OR preventieinstitut[All Fields] OR preventiemaatregel[All Fields] OR preventiemogelijkheden[All Fields] OR preventieparadox[All Fields] OR preventieprogramma[All Fields] OR preventieprogramma's[All Fields] OR preventieproject[All Fields] OR preventies[All Fields] OR preventiestrategie[All Fields] OR preventiestudies[All Fields] OR preventieve[All Fields] OR preventif[All Fields] OR preventif'[All Fields] OR preventifs[All Fields] OR preventig[All Fields] OR preventign[All Fields] OR preventiion[All Fields] OR preventiivisen[All Fields] OR preventiivisesta[All Fields] OR preventiivitekniikasta[All Fields] OR preventilated[All Fields] OR preventilation[All Fields] OR preventilator[All Fields] OR preventilatory[All Fields] OR preventile[All Fields] OR preventimn[All Fields] OR preventin[All Fields] OR preventina[All Fields] OR preventine[All Fields] OR preventing[All Fields] OR preventing'[All Fields] OR preventing''[All Fields] OR preventingchronic[All Fields] OR preventingfasd[All Fields] OR preventings[All Fields] OR preventingthe[All Fields] OR preventingthem[All Fields] OR preventingtroilus[All Fields] OR preventingvarious[All Fields] OR preventinmaternal[All Fields] OR preventino[All Fields] OR preventinve[All Fields] OR preventinych[All Fields] OR preventio[All Fields] OR preventioin[All Fields] OR preventioja[All Fields] OR preventiojanak[All Fields] OR preventiojarol[All Fields] OR preventiom[All Fields] OR prevention[All Fields] OR prevention'[All Fields] OR prevention's[All Fields] OR prevention,[All Fields] OR preventiona[All Fields] OR preventionactive[All Fields] OR preventional[All Fields] OR preventionala[All Fields] OR preventionale[All Fields] OR preventionally[All Fields] OR preventionand[All Fields] OR preventionandcontrol[All Fields] OR preventionas[All Fields] OR preventioncdc[All Fields] OR preventioncontrol[All Fields] OR preventiondes[All Fields] OR preventione[All Fields] OR preventioneffects[All Fields] OR preventionen[All Fields] OR preventionens[All Fields] OR preventiong[All Fields] OR preventiongenetics[All Fields] OR preventioninstitute[All Fields] OR preventionist[All Fields] OR preventionist's[All Fields] OR preventionistic[All Fields] OR preventionistituto[All Fields] OR preventionists[All Fields] OR preventionists'[All Fields] OR preventionl[All Fields] OR preventiono[All Fields] OR preventionoduites[All Fields] OR preventionof[All Fields] OR preventionp[All Fields] OR preventionpluswellness[All Fields] OR preventionpppm[All Fields] OR preventionrecommended[All Fields] OR preventionresearch[All Fields] OR preventions[All Fields] OR preventions'[All Fields] OR preventionsarbetet[All Fields] OR preventionsinstrumenten[All Fields] OR preventionsmentalitet[All Fields] OR preventionsource[All Fields] OR preventionsprogram[All Fields] OR preventionsprojekt[All Fields] OR preventionsuccessful[All Fields] OR preventiont[All Fields] OR preventionworksar[All Fields] OR preventiora[All Fields] OR preventis[All Fields] OR preventitamente[All Fields] OR preventitious[All Fields] OR preventitive[All Fields] OR preventitively[All Fields] OR preventitve[All Fields] OR preventiuve[All Fields] OR preventiv[All Fields] OR preventiva[All Fields] OR preventiva'[All Fields] OR preventivamente[All Fields] OR preventivania[All Fields] OR preventivas[All Fields] OR preventivatahletter[All Fields] OR preventivay[All Fields] OR preventive[All Fields] OR preventive'[All Fields] OR preventivecare[All Fields] OR preventivecounseling[All Fields] OR preventivedentistry[All Fields] OR preventiveim[All Fields] OR preventiveimaging[All Fields] OR preventivelifestyle[All Fields] OR preventively[All Fields] OR preventively'[All Fields] OR preventivemeasures[All Fields] OR preventivemedicine[All Fields] OR preventivement[All Fields] OR preventivenutrition[All Fields] OR preventiveped[All Fields] OR preventives[All Fields] OR preventiveservices[All Fields] OR preventivi[All Fields] OR preventivina[All Fields] OR preventiving[All Fields] OR preventivini[All Fields] OR preventivlagen[All Fields] OR preventivmedel[All Fields] OR preventivmedels[All Fields] OR preventivmedelsanvandning[All Fields] OR preventivmedelsforsaljningen[All Fields] OR preventivmedelsfragan[All Fields] OR preventivmedelsinformation[All Fields] OR preventivmedelskontrollen[All Fields] OR preventivmedelsradgivande[All Fields] OR preventivmedelsradgivning[All Fields] OR preventivmedelsradgivningen[All Fields] OR preventivmedelssokande[All Fields] OR preventivmedicinen[All Fields] OR preventivmedicinsk[All Fields] OR preventivmedlen[All Fields] OR preventivmedlens[All Fields] OR preventivmetod[All Fields] OR preventivmetoder[All Fields] OR preventivn[All Fields] OR preventivn'e[All Fields] OR preventivn'i[All Fields] OR preventivn'ich[All Fields] OR preventivn'iho[All Fields] OR preventivna[All Fields] OR preventivnach[All Fields] OR preventivnaho[All Fields] OR preventivnaia[All Fields] OR preventivne[All Fields] OR preventivnega[All Fields] OR preventivneho[All Fields] OR preventivnej[All Fields] OR preventivni[All Fields] OR preventivnich[All Fields] OR preventivnih[All Fields] OR preventivniho[All Fields] OR preventivniich[All Fields] OR preventivnim[All Fields] OR preventivnimu[All Fields] OR preventivnipece[All Fields] OR preventivno[All Fields] OR preventivnoe[All Fields] OR preventivnog[All Fields] OR preventivnogo[All Fields] OR preventivnoi[All Fields] OR preventivnoj[All Fields] OR preventivnom[All Fields] OR preventivnomedicinske[All Fields] OR preventivnomedicinski[All Fields] OR preventivnomedicinskih[All Fields] OR preventivnomedicinskoj[All Fields] OR preventivnomedicinsku[All Fields] OR preventivnu[All Fields] OR preventivny[All Fields] OR preventivnych[All Fields] OR preventivnye[All Fields] OR preventivnyi[All Fields] OR preventivnykh[All Fields] OR preventivnym[All Fields] OR preventivnymi[All Fields] OR preventivo[All Fields] OR preventivos[All Fields] OR preventivpiller[All Fields] OR preventivradgivningen[All Fields] OR preventivt[All Fields] OR preventivtabletter[All Fields] OR preventivteknik[All Fields] OR preventivu[All Fields] OR preventix[All Fields] OR preventiya[All Fields] OR preventiye[All Fields] OR prevently[All Fields] OR preventment[All Fields] OR preventmmp[All Fields] OR prevento[All Fields] OR preventodontic[All Fields] OR preventodontica[All Fields] OR preventodontiche[All Fields] OR preventodontico[All Fields] OR preventodontics[All Fields] OR preventodontie[All Fields] OR preventodontist[All Fields] OR preventodonzia[All Fields] OR preventol[All Fields] OR preventologickymi[All Fields] OR preventologie[All Fields] OR preventologists[All Fields] OR preventologues[All Fields] OR preventon[All Fields] OR preventor[All Fields] OR preventori[All Fields] OR preventoria[All Fields] OR preventorial[All Fields] OR preventoriale[All Fields] OR preventoriali[All Fields] OR preventorio[All Fields] OR preventorios[All Fields] OR preventoriu[All Fields] OR preventoriul[All Fields] OR preventorium[All Fields] OR preventorium's[All Fields] OR preventoriums[All Fields] OR preventorizarii[All Fields] OR preventors[All Fields] OR preventory[All Fields] OR preventproducts[All Fields] OR preventral[All Fields] OR preventre[All Fields] OR preventriculaire[All Fields] OR preventricular[All Fields] OR preventricularis[All Fields] OR preventriculography[All Fields] OR preventriculostomy[All Fields] OR preventriculus[All Fields] OR preventron[All Fields] OR prevents[All Fields] OR preventsii[All Fields] OR preventsiia[All Fields] OR preventsiiata[All Fields] OR preventspenicillium[All Fields] OR preventting[All Fields] OR preventtivnej[All Fields] OR preventure[All Fields] OR preventx[All Fields] OR preventyvna[All Fields] OR preventza[All Fields])) AND (("economics"[Subheading] OR "economics"[All Fields] OR "economics"[MeSH Terms]) OR "economic evaluation"[TIAB] OR "cost effectiveness"[All Fields] OR "hospital expenditure"[All Fields] OR "healthcare costs"[All Fields] OR "cost analysis"[All Fields] OR "costs and cost analysis"[TIAB])) AND preoperative[All Fields]) AND ("antibiotic prophylaxis"[MeSH Terms] OR ("antibiotic"[All Fields] AND "prophylaxis"[All Fields]) OR "antibiotic prophylaxis"[All Fields]) AND "adult"[MeSH Terms] | 344 |
| CINAHL;  1980 to 2017 | Cumulative Index to Nursing and Allied Health Literature |  | (MH "Cost Benefit Analysis") OR (MH "Costs and Cost Analysis") OR (MH "Economics") OR (MH "Health Care Costs") OR "economics OR cost effectiveness" AND (MH "Surgical Wound Infection") OR (MH "Surgical Site") OR (MH "Surgical Wound") OR "Surgical site infection* OR surgical wound infection* OR postoperative wound infection" AND (MH "Antibiotic Prophylaxis") OR "antibiotic prophylaxis". | 38 |
|  |  | #6 | #1 AND #4 AND #5 | 38 |
|  |  | #5 | (MH "Antibiotic Prophylaxis") OR "antibiotic prophylaxis" ) OR antibiotic prophylac* | 3,989 |
|  |  | #4 | #2 OR #3 | 6,523 |
|  |  | #3 | (MH "Surgical Wound Infection") OR (MH "Surgical Site") OR (MH "Surgical Wound") OR "Surgical site infection* OR surgical wound infection* OR postoperative wound infection" | 6,249 |
|  |  | #2 | (MH "Surgical Wound Infection") OR (MH "Surgical Site") OR "surgical site infection" | 5,848 |
|  |  | #1 | (MH "Cost Benefit Analysis") OR (MH "Costs and Cost Analysis") OR (MH "Economics") OR (MH "Health Care Costs") OR "economics OR cost effectiveness | 54,554 |
| WOS;  1970 to 2017 | Web of Science, core collection |  | TS=(economics OR cost effectiveness) OR TS=(Cost benefit analysis) OR TS=(cost and cost analysis) OR TS=(health care costs) *DocType=All document types; Language=All languages;* AND TOPIC: (surgical site infection) *OR* TOPIC: (surgical site) *OR* TOPIC: (surgical wound infection) *OR* TOPIC: (surgical wound infection*) *OR* TOPIC: (surgical site infection*) *OR* TOPIC: (postoperative wound infection*) *DocType=All document types; Language=All languages;* AND TOPIC: (antibiotic prophylaxis) AND TOPIC: (adult) *DocType=All document types; Language=All languages;* | 51 |
|  |  | #5 | #4 AND #3 AND #2 AND #1  *DocType=All document types; Language=All languages; limited to 1970 to 2017* | [51](http://apps.webofknowledge.com.ezproxy.library.uq.edu.au/summary.do?product=WOS&doc=1&qid=5&SID=Z2OWrHZVTAK2rVZxs65&search_mode=CombineSearches&update_back2search_link_param=yes) |
|  |  | #4 | TOPIC: ("antibiotic prophylaxis" OR "antibiotic" AND "prophylaxis" OR "antibiotic prophylaxis" OR antimicrobial OR "Anti-Infective Agents")  *DocType=All document types; Language=All languages; limited to 1970 to 2017* | [185,439](http://apps.webofknowledge.com.ezproxy.library.uq.edu.au/summary.do?product=WOS&doc=1&qid=4&SID=Z2OWrHZVTAK2rVZxs65&search_mode=GeneralSearch&update_back2search_link_param=yes) |
|  |  | #3 | TOPIC: ("economics" OR "economics" OR "economics" OR "cost effectiveness" OR "hospital expenditure" OR "healthcare costs" OR "cost analysis")  *DocType=All document types; Language=All languages; limited to 1970 to 2017* | [1,301,677](http://apps.webofknowledge.com.ezproxy.library.uq.edu.au/summary.do?product=WOS&doc=1&qid=2&SID=Z2OWrHZVTAK2rVZxs65&search_mode=GeneralSearch&update_back2search_link_param=yes) |
|  |  | #2 | TOPIC: (prevent OR prevent* OR "prevention and control")  *DocType=All document types; Language=All languages; limited to 1970 to 2017* | [1,301,677](http://apps.webofknowledge.com.ezproxy.library.uq.edu.au/summary.do?product=WOS&doc=1&qid=2&SID=Z2OWrHZVTAK2rVZxs65&search_mode=GeneralSearch&update_back2search_link_param=yes) |
|  |  | #1 | TOPIC: (“Surgical Wound Infection" OR "surgical site infection*" OR "surgical wound infection*" OR "postoperative wound infection*")  *DocType=All document types; Language=All languages; limited to 1970 to 2017* | [9,663](http://apps.webofknowledge.com.ezproxy.library.uq.edu.au/summary.do?product=WOS&doc=1&qid=1&SID=Z2OWrHZVTAK2rVZxs65&search_mode=GeneralSearch&update_back2search_link_param=yes) |
| EconLit  1970 to 2017 | Journal of Economic Literature and the Index of Economic Articles |  | economics OR cost analysis OR cost benefit OR cost effectiveness OR health care costs OR cost allocation OR cost AND surgical sites OR surgical site infection OR surgical wound infections OR surgical wound infections prevention OR postoperative wound infection AND antibiotics OR antibiotic prophylaxis. | 11 |
| Dare and NHSEED;  1994 to present | Database of abstracts of reviews of effects (DARE) including the National Institute of health Research [Economic Evaluation Database (NHS EED)](http://www.crd.york.ac.uk/crdweb/) |  | MeSH DESCRIPTOR Economics, Nursing EXPLODE ALL TREES AND MeSH DESCRIPTOR Cost-Benefit Analysis EXPLODE ALL TREES OR MeSH DESCRIPTOR Costs and Cost Analysis EXPLODE ALL TREES OR MeSH DESCRIPTOR Economics, Medical EXPLODE ALL TREES OR MeSH DESCRIPTOR Economics, Hospital EXPLODE ALL TREES OR MeSH DESCRIPTOR Health Care Costs EXPLODE ALL TREES OR (cost effectiveness) AND MeSH DESCRIPTOR Surgical Wound Infection EXPLODE ALL TREES OR MeSH DESCRIPTOR Infection Control EXPLODE ALL TREES OR (surgical site infection) OR (surgical wound infection) OR (post operative wound infection) AND MeSH DESCRIPTOR Antibiotic Prophylaxis EXPLODE ALL TREES | 48 |
| Cochrane Central Register of Controlled Trials;  1996 to 2017 | Cochrane Library |  | "surgical site infection" or "surgical wound infection" or "surgical site" or "post operative" or postoperative wound infection in Title, Abstract, Keywords and "economics" or "cost effectiveness" or "cost benefit analysis" or "cost analysis" or "cost" in Title, Abstract, Keywords and "antibio-prophylaxis" or antibiotic prophylaxis in Title, Abstract, Keywords and "adult" in Title, Abstract, Keywords (Word variations have been searched) | 136 |

**Table S2** List of OECD countries*

| Australia | Greece | Poland |
| --- | --- | --- |
| Austria | Iceland | Portugal |
| Belgium | Ireland | Slovak Republic |
| Canada | Italy | Slovenia |
| Chile | Israel | Spain |
| Czech Republic | Japan | Sweden |
| Denmark | Korea, Rep. | Switzerland |
| Estonia | Luxembourg | United Kingdom |
| Finland | Netherlands | United States |
| France | New Zealand |  |
| Germany | Norway |  |
| **OECD,** Organisation for Economic Co-operation and Development  ***REF: 28 -** World Bank Group. Data: Country and Lending Groups 2014 [cited 16 July 2014]. Available from: <http://data.worldbank.org/about/country-and-lending-groups>. | | |

**Table S3** Characteristics of included studies

| Reference | Country with years of study | Type of study | Study duration (years) | Population | Follow-up | Preoperative prophylaxis | | Preoperative prophylaxis outcome measures | | Conclusion |
| --- | --- | --- | --- | --- | --- | --- | --- | --- | --- | --- |
|  |  |  |  |  |  | Control | Intervention | Primary (efficacy) | Secondary (cost analysis) |  |
| Blair *et al.*^35^ (1995) | Greece (1976–1989) | Retrospective chart review | 15 | ‘Clean’ neck dissection: 192 | n.s. | No prophylaxis | Cefazolin 600 mg* | First-generation cephalosporin; clindamycin and penicillin *versus* no antibiotic to prevent postoperative wound infection | Cost-benefit analysis (hospital stay and cost) | No significant difference in infections. Preoperative antibiotic prophylaxis advocated. Cost-effective |
|  |  |  |  |  |  | No prophylaxis | Clindamycin 2 g* |  |  |  |
|  |  |  |  |  |  | No prophylaxis | Penicillin* |  |  |  |
|  |  |  |  |  |  | No prophylaxis | Drug name n.s.*† |  |  |  |
| Bold *et al.*^36^ (1998) | USA (n.s.) | Double-blind RCT | 5.25 | Axillary lymph node dissection: 178 | 4 weeks after surgery | Placebo( normal saline) | Cefonicid 1 g (single dose) | Second-generation cephalosporin *versus* placebo to decrease postoperative wound complications | Cost-benefit analysis | No significant difference in infections. Preoperative antibiotic prophylaxis advocated |
| Davey *et al.*^37^ (1988) | Scotland (n.s.) | Double-blind RCT | n.s. | Abdominal or vaginal hysterectomy: 400 | Every 3 days, then postdischarge (visit week 2, phone call week 6) | Placebo (normal saline) | Cephradine 2 g (single dose) | First-generation cephalosporin *versus* broad-spectrum penicillin to prevent wound infection | Cost-benefit analysis (patient, hospital and community services) | Cephradine antibiotic prophylaxis advocated in abdominal hysterectomy. Antibiotic prophylaxis questionable in vaginal hysterectomy |
|  |  |  |  |  |  |  | Mezlocillin 5 g (single dose) |  |  |  |
| Dhadwal *et al.*^38^ (2007) | UK (2003–2004) | Double-blind RCT | 1 | Median sternotomy for primary CABG of at least 1 thoracic artery and at least 1 of 4 defined risk factors: 201‡ and 186§ | Daily until discharge, then postdischarge (week 6 and 90 days) | Cefuroxime 1.5 g (single dose), then cefuroxime 750 mg at reversal of anticoagulation, 8 and 16 h after surgery | Rifampicin 600 mg (single dose), then gentamicin 2 mg/kg + vancomycin 15 mg/kg on induction of anaesthesia. Postoperative vancomycin 7.5 mg/kg at 12, 24 and 36 h | Second-generation♯ cephalosporin *versus* gentamicin combined with rifampicin and vancomycin to prevent sternal wound infection | Cost-benefit analysis | Longer and broader-spectrum preoperative antibiotic prophylaxis advocated. Cost-effective |
| Dijksman *et al.*^39^ (2012) | The Netherlands (2003–2008) | Cost study nested in double-blind RCT[^47^](#_ENREF_6) | 5 | Intestinal resection with primary anastomosis, with or without a diverting ileostomy or closure of a temporary colostomy: 289 | 1 year | Placebo for 2 days before surgery, then parenteral perioperative cefuroxime 1500 mg + metronidazole 500 mg 30 min before surgery. Cefuroxime 1500 mg + metronidazole 500 mg continued 8-hourly for 24 h | SDD (polymyxin B sulphate100 mg + tobramycin 80mg + amphotericin B 500 mg) for 2 days before surgery and continued for at least 3 days after surgery or until normal bowel function. Parenteral perioperative antibiotic cefuroxime 1500 mg + metronidazole 500 mg 30 min before surgery. Cefuroxime 1500 mg + metronidazole 500 mg continued 8-hourly for 24 h | Perioperative selective decontamination of digestive tract (polymyxin B sulphate with tobramycin and amphotericin B) *versus* placebo to reduce infection | Cost-effectiveness analysis | Selective decontamination of digestive tract advocated. Cost-effective |
| Garcia-Rodriguez *et al.*^40^ (1989) | Spain (1987–1988) | Open RCT | 1 | Gastroduodenal or biliary surgery with at least 1 of 11 defined risk factors: 1451 | 16 days | Cefoxitin 2 g (single i.v. dose), then cefoxitin 2 g 6,12 and 18 h after surgery | Cefotaxime 1 g (single dose) | Second- and third-generation cephalosporin♯ to prevent postoperative infection | Cost-benefit analysis | Cefotaxime antibiotic prophylaxis advocated. Cost-effective |
| Jones *et al.*^41^ (1987) | USA (1984–1985) | Observer-blinded RCT¶ | 1 | Obstetrics and gynaecology, gastrointestinal; orthopaedics and other (total joint replacement and open reduction of fractures) surgical procedures: 812 | 30 days | Cefotaxime 1.0 g (slow i.v. bolus after anaesthesia but 30 min before incision). Additional cefotaxime 1.0 g given during surgery if procedure duration 2 h or more. For bowel surgery, standard bowel preparation before prophylaxis | Cefoperazone 1.0 g (slow i.v. bolus after anaesthesia but 30 min before incision). For bowel surgery, standard bowel preparation before prophylaxis | Two third-generation cephalosporins to prevent perioperative infection | Cost containment | Both cefoperazone and cefotaxime antibiotic prophylaxis advocated. Both cost-effective |
| Marroni *et al.*^42^ (1999) | Italy (1995–1997) | Double-blind RCT | 2 | Abdominal aortic or lower limb prosthetic vascular surgery: 238 | Daily until discharge, then postdischarge (3 monthly for 1 year, then at 24 months) | Cefazolin 2 g (single i.v. dose) | Teicoplanin 400 mg (single dose) | Efficacy and tolerability of first-generation cephalosporin and a glycopeptide to prevent postoperative infection | Cost-benefit analysis | Cefazolin antibiotic prophylaxis advocated. Cost-effective |
| Matkaris *et al.*^43^ (1991) | Greece (n.s.) | Blinded RCT | n.s. | Abdominal hysterectomy: 200 | 4–5 days if no SSI, otherwise kept in hospital until infection resolved | No prophylaxis | Ceftriaxone 2 g (single dose). Additional dose if postoperative infection | Efficacy and safety of three third-generation cephalosporins to prevent postoperative infection | Cost-benefit analysis | Single dose of any of the three antibiotic prophylaxes advocated. Cefotaxime was most cost-effective |
|  |  |  |  |  |  |  | Cefotaxime 2 g (single dose). Additional dose if postoperative infection |  |  |  |
|  |  |  |  |  |  |  | Ceftazidime 2 g (single dose). Additional dose if postoperative infection |  |  |  |
| Matsui *et al.*^44^ (2014) | Japan (2007–2013) | Observer-blinded RCT¶ | 6 | Laparoscopic cholecystectomy for gallbladder stones or polyps: 437 | 8 days postsurgery in outpatient setting | No prophylaxis | Cefazolin 1 g (3 doses before skin incision, then 12 and 24 h after surgery). Additional cefazolin 1 g in theatre if duration of surgery more than 3 h | First-generation† cephalosporin to reduce postoperative complications, including SSI and distant infection | Cost-effectiveness analysis | Antibiotic prophylaxis advocated. Cost-effective |
| Sisto *et al.*^45^ (1994) | Finland (1993) | RCT# | 1 | CABG: 551 | Daily until discharge (10–12 days) or to another hospital (6–7 days) | Ceftriaxone 2 g (single dose) | Cefuroxime 1.5 g (single dose), then cefuroxime 1.5 g (8-hourly to end of postoperative day 2) | Efficacy and side-effects of single-dose third-generation cephalosporin *versus* multiple doses of second-generation cephalosporin to prevent postoperative infection | Cost-benefit analysis | Efficacy of ceftriaxone and cefuroxime equivalent. Ceftriaxone cheaper and simpler to use |
| Wilson *et al.*^46^ (2008) | USA (2002–2005) | Cost study nested in double-blind RCT[^48^](#_ENREF_15) | 3 | Colorectal surgery: 672** | 4 weeks after surgery | Ertapenem 1 g (single dose) | Cefotetan 2 g (single dose) | Preoperative prophylaxis of second-generation cephalosporin and a β-lactam to reduce postoperative infectious complications | Cost-benefit analysis | Ertapenem antibiotic prophylaxis advocated. Cost-effective |

*Prophylactic antibiotic dose not stated; †antibiotic trade name or generation of the cephalosporin not stated; ‡intention-to-treat data for antibiotic efficacy; §per-protocol data for costs^32^; ¶clinicians doing postoperative assessments and follow-up blinded to randomization codes; #blinding not stated; **per-protocol data. n.s., Not stated; CABG, coronary artery bypass graft; SDD, selective decontamination of digestive tract; i.v., intravenous; SSI, surgical-site infection.

**Table S4** CHEERS checklist of reporting quality

|  | **Questions** | **Blair**  **1995**  **[35]** | **Bold**  **1998**  **[36]** | **Davey**  **1988**  **[37]** | **Dhadwal**  **2007**  **[38]** | **Dijksman**  **2012**  **[39]** | **Garcia-Rodriguez**  **1989**  **[40]** | **Jones**  **1987**  **[41]** | **Marroni**  **1999**  **[42]** | **Matkaris**  **1991**  **[43]** | **Matsui**  **2014**  **[44]** | **Sisto**  **1994**  **[45]** | **Wilson**  **2008**  **[46]** |
| --- | --- | --- | --- | --- | --- | --- | --- | --- | --- | --- | --- | --- | --- |
| Title and abstract | Title | 1 | 0 | 2 | 2 | 2 | 0 | 0 | 0 | 1 | 0 | 0 | 1 |
|  | Abstract | 2 | 2 | 1 | 2 | 2 | 1 | 1 | 1 | 1 | 2 | 2 | 1 |
| Introduction | Background and objectives | 2 | 2 | 2 | 2 | 2 | 2 | 1 | 2 | 2 | 2 | 2 | 1 |
| Methods | Target population and etc | 2 | 2 | 2 | 2 | 2 | 1 | 2 | 1 | 2 | 2 | 2 | 1 |
|  | Setting and location | 2 | 2 | 2 | 2 | 2 | 1 | 1 | 1 | 2 | 2 | 2 | 1 |
|  | Study perspective | 2 | 2 | 2 | 1 | 2 | 1 | 1 | 1 | 2 | 2 | 2 | 1 |
|  | Comparators | 2 | 2 | 2 | 1 | 2 | 1 | 1 | 1 | 2 | 2 | 2 | 1 |
|  | Time horizon | 0 | 2 | 0 | 1 | 2 | 1 | 1 | 1 | 0 | 1 | 2 | 1 |
|  | Discount rate | N/A | N/A | N/A | N/A | N/A | N/A | N/A | N/A | N/A | N/A | N/A | N/A |
|  | Choice of health outcomes | 0 | 2 | 1 | 2 | 2 | 1 | 1 | 1 | 0 | 1 | 1 | 1 |
|  | Measurement of effectiveness | 0 | 2 | 1 | 2 | 2 | 1 | 1 | 1 | 0 | 1 | 1 | 1 |
|  | Measurement and valuation of preferences etc. | N/A | 2 | 2 | 2 | 2 | 1 | 1 | 1 | 1 | 1 | 1 | 1 |
|  | Estimating resources etc. | N/A | 2 | 0 | 2 | 2 | 1 | 1 | 1 | 1 | 1 | 1 | 1 |
|  | Currency, price date etc. | 1 | 0 | 0 | 0 | 2 | 1 | 1 | 0 | 1 | 1 | 0 | 1 |
|  | Choice of model | N/A | N/A | N/A | N/A | N/A | N/A | N/A | N/A | N/A | N/A | N/A | N/A |
|  | Assumptions | N/A | N/A | N/A | N/A | N/A | N/A | N/A | N/A | N/A | N/A | N/A | N/A |
|  | Analytical methods | 1 | 1 | 1 | 1 | 2 | 1 | 1 | 1 | 1 | 1 | 1 | 1 |
| Results | Study parameters | 0 | 0 | 0 | 1 | 2 | 0 | 1 | 1 | 1 | 1 | 1 | 1 |
|  | Incremental costs and outcomes | 0 | 0 | 0 | 0 | 2 | 2 | 0 | 0 | 0 | 0 | 0 | 0 |
|  | Characterizing uncertainty | 0 | 0 | 0 | 0 | 2 | 0 | 0 | 0 | 0 | 0 | 1 | 2 |
|  | Characterizing heterogeneity | 0 | 0 | 1 | 2 | 1 | 1 | 1 | 1 | 0 | 1 | 1 | 1 |
| Discussion | Study findings, limitations, etc | 1 | 2 | 1 | 2 | 2 | 1 | 1 | 1 | 1 | 1 | 1 | 1 |
| Other | Source of funding | 2 | 2 | 0 | 0 | 0 | 0 | 2 | 0 | 0 | 2 | 0 | 0 |
|  | Conflicts of interest | 0 | 0 | 0 | 2 | 0 | 0 | 0 | 0 | 0 | 2 | 0 | 0 |
| Overall score for 24 questions | | 7/24 | 13/24  M  L 24 | 7/24  L  L /24 | 11/24  L  L 4 | 18/24  H  L 8/24 | 2/24  L  L 8/24 | 2/24  L  4 | 1/24  L  /24 | 6/24  L  24 | 8/24  L  8/24 | 7/24  L  /24 | 1/24  L  24 |
| Overall score for 21 questions (excluding: discount rate, choice of model &assumptions) | | 7/21 | 13/21  M  L 24 | 7/21  L  L /24 | 11/21  L  L 4 | 18/21  H  L 8/24 | 2/21  L  L 8/24 | 2/21  L  4 | 1/21  L  /24 | 6/21  L  24 | 8/21  L  8/24 | 7/21  L  /24 | 1/21  L  24 |
| N/A = not applicable  0 = not reported, 1 = poorly reported, 2 = well reported | | | | | | | | | | | | | |

**Table S5** Quality assessment checklist for assessing economic evaluations of included studies

| **First author, year [reference number]** | | | | | | | | | | | | | | | | | | | | | | | | | | |
| --- | --- | --- | --- | --- | --- | --- | --- | --- | --- | --- | --- | --- | --- | --- | --- | --- | --- | --- | --- | --- | --- | --- | --- | --- | --- | --- |
|  | **Questions** | **Blair**  **1995**  **[35]** | | **Bold**  **1998**  **[36]** | | **Davey**  **1988**  **[37]** | | **Dhadwal**  **2007**  **[38]** | | **Dijksman**  **2012**  **[39]** | | **Garcia-Rodriguez**  **1989**  **[40]** | | **Jones**  **1987**  **[41]** | | **Marroni**  **1999**  **[42]** | | **Matkaris**  **1991**  **[43]** | | **Matsui**  **2014**  **[44]** | | **Sisto**  **1994**  **[45]** | | **Wilson**  **2008**  **[46]** | |  |
| **1** | Well defined question stated? | Yes | | Yes | | Yes | | Yes | | Yes | | Yes | | Yes | | Yes | | Yes | | Yes | | Yes | | Yes | |  |
| **2** | Description of alternatives? | Yes | | Yes | | Yes | | Yes | | Yes | | Yes | | Yes | | Yes | | Yes | | Yes | | Yes | | Yes | |  |
| **3** | Evidence of clinical effectiveness established? | Yes | | Yes | | Unsure | | Yes | | Yes | | Yes | | Yes | | Yes | | Yes | | Yes | | No | | Yes | |  |
| **4** | Relevant costs and outcomes identified? | No | | Yes | | No | | No | | Yes | | Yes | | Yes | | No | | Yes | | No | | Yes | | Yes | |  |
| **5a** | Costs measured accurately in appropriate units? | No | | No | | Yes | | Yes | | Yes | | Yes | | Yes | | No | | No | | Yes | | Yes | | Yes | |  |
| **5b** | Outcomes measured accurately in appropriate units | No | | No | | Yes | | Yes | | Yes | | Yes | | Yes | | No | | No | | Yes | | Yes | | Yes | |  |
| **6a** | Costs valued credibly? | No | | Yes | | Yes | | Yes | | Yes | | Yes | | Yes | | No | | Yes | | Yes | | Yes | | Yes | |  |
| **6b** | Outcomes valued credibly? | No | | Yes | | Yes | | Yes | | Yes | | Yes | | Yes | | No | | Yes | | Yes | | Yes | | Yes | |  |
| **7** | Costs discounted? | No | | No | | N/A | | No | | N/A | | No | | N/A | | No | | No | | N/A | | N/A | | N/A | |  |
| **8** | Was incremental analysis performed? | No | | No | | No | | No | | Yes | | No | | No | | No | | No | | No | | No | | No | |  |
| **9** | Was sensitivity analysis performed? | No | | No | | No | | No | | Yes | | No | | No | | No | | No | | No | | No | | No | |  |
| **10** | Was generalisability discussed? | No | | Yes | | Yes | | Yes | | Yes | | Yes | | Yes | | Yes | | No | | Yes | | Yes | | Yes | |  |
| **Overall study quality** | | | 3/12  L/U | | 7/12  M/A | | 7/12  M/A | | 8/12  M/A | | 11/12  H | | 9/12  H | | 9/12  H | | 4/12  L/U | | 6/12  M/A | | 8/12  M/A | | 8/12  M/A | | 9/12  H | |
| **H = High; L/U= Low/Unacceptable; M/A= Moderate/Acceptable; N/A = Not applicable** | | | | | | | | | | | | | | | | | | | | | | | | | | |

**Table S6** Summary of reported costs and incremental cost effectiveness ratio calculated from study data

| Reference | Cost analysis | Source of cost data, with year and currency | Intervention *versus* control | *n* | Intervention  failure* | Control failure* | Treatment effect (TEc − TEi) | Mean cost of intervention (includes treatment cost) | Mean cost of control (includes treatment cost) | Incremental cost per patient | Incremental cost per patient (2016 €)† | ICER (2016 €)† |
| --- | --- | --- | --- | --- | --- | --- | --- | --- | --- | --- | --- | --- |
| Blair *et al.*^35^‡ | Cost-benefit analysis (hospital stay and cost) | Hospital bills used to establish per diem charge (1992; US $) | Cefazolin, clindamycin and cefoperazone *versus* placebo | 192 | 3 of 93 (3) | 10 of 99 (10) | 7 | $36 240.00 | $36 030.00 | $210.00 | 293.79 | Dominant |
| Bold *et al.*^36^§ | Cost-benefit analysis | n.s. (1998; US $) | Cefonicid *versus* placebo | 178 | 5 of 88 (6) | 12 of 90 (13) | 7 | $149.80 | $364.87 | −$215.07 | −269.26 | Dominant |
| Davey *et al.*^37^¶ | Cost-benefit analysis (patient, hospital and community services) | Antibiotic costs calculated from MMIS and GP prescription (1988; £ sterling) | AH: cephradine *versus* placebo | 300 | 40 of 97 (41) | 53 of 102 (52.0) | 11 | £18.26 | £31.34 | −£13.08 | −37.92 | Dominant |
|  |  |  | AH: mezlocillin *versus* placebo |  | 40 of 101 (39.6) | 53 of 102 (52.0) | 12.4 | £17.61 | £31.34 | −£13.73 | −37.92 | Dominant |
|  |  |  | VH: cephradine *versus* placebo | 100 | 14 of 34 (41) | 15 of 29 (52) | 11 | £40.60 | £41.20 | −£0.60 | −1.65 | Dominant |
|  |  |  | VH: mezlocillin *versus* placebo |  | 7 of 37 (19) | 15 of 29 (52) | 33 | £8.80 | £41.20 | −£32.40 | −89.50 | Dominant |
| Dhadwal *et al.*^38^# | Cost-benefit analysis | Hospital acquisition costs, from hospital accountant (2004; US $) | Rifampicin +gentamicin +vancomycin *versus* cefuroxime | 186 | 8 of 87 (9) | 25 of 99 (25) | 16 | $15 158.00 | $19,054.00 | −$3896.00 | −4315.99 | Dominant |
| Dijksman *et al.*^39^** | Cost-effectiveness analysis | Hospital ledger (2008; €) | SDD (amphotericin B, polymyxin B sulphate + tobramycin) *versus* placebo | 289 | 28 of 143 (19.6) | 45 of 146 (30.8) | 11.2 | €12 031.00 | €14 635.00 | −€2604.00 | −2731.28 | Dominant |
| Garcia-Rodriguez *et al.*^40^†† | Cost-benefit analysis | Hospital costs at time of trial (1987–1988) | Cefotaxime *versus* cefoxitin | 1451 | 22 of 722 (3.3) | 54 of 716 (7.7) | 4.4 | $28.64 | $104.43 | −$75.79 | −120.72 | Dominant |
| Jones *et al.*^41^‡‡ | Cost containment | n.s. (1987; US $) | Cefoperazone *versus* cefotaxime | 812 | 9 of 411 (2.2) | 12 of 401 (3.0) | 0.8 | $14.50 | $12.90 | $1.60 | 2.64 | 5.12 |
| Marroni *et al.*^42^§§ | Cost-benefit analysis | n.s. (1998; US $) | Cefazolin *versus* teicoplanin | 238 | 7 of 119 (5.9) | 2 of 119 (1.7) | −4.2 | $4803.13 | $4361.86 | $441.27 | 552.45 | Dominated by control |
| Matkaris *et al.*^43^¶¶ | Cost-benefit analysis | n.s. (1991; drachma converted to US $) | Ceftriaxone *versus* no antibiotic | 200 | 3 of 50 (6) | 15 of 50 (30) | 24 | $150.12 | $248.03 | −$97.91 | −140.10 | Dominant |
|  |  |  | Cefotaxime *versus* no antibiotic |  | 4 of 50 (8) | 15 of 50 (30) | 22 | $128.06 | $248.03 | −$119.97 | −171.67 | Dominant |
|  |  |  | Ceftazidime *versus* no antibiotic |  | 4 of 50 (8) | 15 of 50 (30) | 22 | $137.81 | $248.03 | −$110.22 | −157.71 | Dominant |
| Matsui *et al.*^44^## | Cost-effectiveness analysis | Costs according to the National Health Insurance System (2013 ; US $) | Cefazolin *versus* no antibiotic | 1037 | 6 of 518 (1.2) | 35 of 519 (6.7) | 5.5 | $766.10 | $831.90 | −$65.80 | −60.75 | Dominant |
| Sisto *et al.*^45^*** | Cost-benefit analysis | Treatment, purchase and delivery costs (1994; US $) | Ceftriaxone *versus* cefuroxime | 551 | 21 of 274 (7.7) | 23 of 277 (8.3) | 0.6 | $36.11 | $107.82 | −$71.71 | −95.95 | Dominant |
| Wilson *et al.*^46^††† | Cost-benefit analysis | Hospital claims database (2005 US $ from Premier Perspective Comparative Database) | Ertapenen *versus* cefotetan | 672 | 143 of 334 (42.8) | 95 of 338 (28.1) | −14.7 | $15 230.00 | $17 411.00 | −$2181.00 | −2340.81 | Dominant |

*Values in parentheses are percentages. †‘Discounted’ cost per patient and, incremental cost-effectiveness ratio (ICER) calculated by means of a two-step discounting process using the Campbell and Cochrane Economics Methods Group–Evidence for Policy and Practice Information and Coordinating Centre cost converter web-based tool^32,33^. The 2016 implied conversion factor is US $1 = £0.70 sterling; the 2016 euro conversion factor is £1 sterling = €1.28. ‡Treatment effects of cefazolin, clindamycin and cefoperazone were pooled, and costs were pooled and averaged; cost inferred from study setting to be US$; for conversion of 1992 US dollars to 2016 British pounds, the implied inflation factor for US $1 in 1992 to 2016 value is 1.57. §Price year inferred from publication date; for conversion of 1998 US dollars to 2016 British pounds, the implied inflation factor for US $1 in1998 to 2016 is 1.41. ¶Price year inferred from publication date; for conversion of 1988 British pounds to 2016 British pounds, the implied inflation factor for £1 sterling in1988 to 2016 is 2.16. #Price year inferred from study end date; cost data based on per-protocol analysis; for conversion of 2004 US dollars to 2016 British pounds, the implied inflation factor for US $1 in 2004 to 2016 is 1.24. **For conversion of 2008 euros to 2016 euros, the implied inflation factor for €1 in 2008 to 2016 is 1.05. ††Cost inferred from study setting to be US $; for conversion of 1988 US dollars to 2016 British pounds, the implied inflation factor for US $1 in 1988 to 2016 is 1.79; infection data were missing for six patients in the control group and seven in the intervention group. ‡‡Price year inferred from publication date; all treatment failures; for conversion of 1987 US dollars to 2016 British pounds, the implied inflation factor for US $1 in 1987 to 2016 is 1.87. §§Price year inferred from study end date; for conversion of 1998 US dollars to 2016 Britsh pounds, the implied inflation factor for US $1 in1998 to 2016 is 1.41. ¶¶Price year inferred from publication date; for conversion of 1991 US dollars to 2016 British pounds, the implied inflation factor for US $1 in 1991 to 2016 is 1.61. ##Price year inferred from publication date; for conversion of 2013 US dollars to 2016 Britsh pounds, ,the implied inflation factor for US $1 in 2013 to 2016 is 1.04. ***Price year inferred from study end date; for conversion of 1994 US dollars to 2016 Britsh pounds, the implied inflation factor for US $1 in 1994 to 2016 is 1.50. †††Cost inferred from study setting to be US$; cost data based on per-protocol analysis; for conversion of 2005 US dollars to 2016 Britsh pounds, the implied inflation factor for US $1 in 2005 to 2016 is 1.21. TEc, treatment effect for control; TEi, treatment effect for intervention; AH, abdominal hysterectomy; VH, vaginal hysterectomy; SDD, selective decontamination of digestive tract
